# ﻿A new species of *Illacme* from southern California (Siphonophorida, Siphonorhinidae)

**DOI:** 10.3897/zookeys.1167.102537

**Published:** 2023-06-21

**Authors:** Paul E. Marek, Charity L. Hall, Cedric Lee, James Bailey, Matt C. Berger, Matt T. Kasson, William Shear

**Affiliations:** 1 Virginia Polytechnic Institute and State University, Department of Entomology, Blacksburg, Virginia 24061, USA Virginia Polytechnic Institute and State University Blacksburg United States of America; 2 Blacksburg, Virginia 24060, USA Unaffiliated Blacksburg United States of America; 3 University of California, Berkeley, Department of Environmental Science, Policy, and Management, California, 94720, Berkeley, USA University of California Berkeley United States of America; 4 Long Beach, California, 90803, USA Unaffiliated Long Beach United States of America; 5 West Virginia University, Division of Plant and Soil Sciences, Morgantown, West Virginia, USA West Virginia University Morgantown United States of America; 6 Hampden-Sydney College, Hampden Sydney, Virginia, USA Hampden-Sydney College Hampden Sydney United States of America

**Keywords:** Colobognatha, *Illacmeplenipes*, interstitial, Myriapoda, *Siphonorhinus*, super-elongation

## Abstract

The millipede fauna inhabiting deep soil are poorly known. They are small and threadlike, slow moving, lacking pigmentation, and rarely encountered due to their obscure underground way of life. One family, the Siphonorhinidae, encompasses four genera and 12 species in a fragmentary distribution in California, southern Africa, Madagascar, the Malay Archipelago, and Indo-Burma. The family is represented in the Western Hemisphere by a single genus, *Illacme* Cook & Loomis, 1928 from California, with its closest known relative, *Nematozoniumfilum* Verhoeff, 1939, from southern Africa. A new species of this family is documented from soil microhabitats in the Los Angeles metropolitan area, *Illacmesocal* Marek & Shear, **sp. nov.** Based on this discovery and the recent documentation of other endogean millipede species, we show that these grossly understudied subterranean fauna represent the next frontier of discovery. However, they are threatened by encroaching human settlement and habitat loss, and conservation of this species and other subterranean fauna is of high importance.

## ﻿Introduction

In 1926, American myriapodologist Orator Cook discovered a millipede with 750 legs under a stone in an oak forest near San Juan Bautista, California. As it had more legs than any other animal known at the time, he and Harold Loomis aptly named this millipede *Illacmeplenipes* Cook & Loomis, 1928, meaning “in highest fulfillment of feet” (Cook and Loomis 1928; Marek and Bond 2006). Six years later, Loomis described a 742-legged millipede, *Siphonophoramillepeda* Loomis, 1934 from Tobago and remarked of an unwitting contest among millipedes for the greatest number of legs (Loomis 1934: 9):

„*The unconscious rivalry between milliped[e]s for the greatest number of segments becomes keener with the finding of this Tobago Island species. Several years ago another species of this order was described from California, one of the specimens having 192 segments [and 750 legs] but the others falling considerably below this number. Although 192 segments is the greatest number thus far known for a milliped[e], it is not unlikely that specimens of the present species or the California one will be found exceeding this number.*“

In 1940, and apparently unaware of the New World taxa, Verhoeff (1940) described the 710-legged millipede *Nematozoniumelongatissimum* Verhoeff, 1940 from Bulwer, South Africa and commented about its unprecedented number of legs (Shelley and Hoffman 2004). Eighty years later, a millipede with a record-breaking 1306 legs, *Eumillipespersephone* Marek, 2021, was discovered 60 meters underground in Western Australia (Marek et al. 2021). With nearly twice the number of legs of *I.plenipes*, *E.persephone* is in a separate order, the Polyzoniida, and in the family Siphonotidae (Marek et al. 2021). Currently, five millipede species are known to have more than 710 legs and possess super-elongated trunks with more than 180 diplosegments (hereafter “rings”): *E.persephone* (1360 legs, 330 rings), family Siphonotidae; *I.plenipes* (750 legs, 192 rings), *Nematozoniumelongatissimum* (710 legs, 182 rings), family Siphonorhinidae; and *S.millepeda* and *Siphonacmelyttoni* Cook & Loomis, 1928 (742 legs, 190 rings), family Siphonophoridae.

Colobognath millipedes emerge from the egg with at least four leg pairs and incrementally add legged-segments during development, even after attaining sexual maturity, a process known as euanamorphosis (Enghoff et al. 1993; Wong et al. 2020). Although these millipedes have an average maximum ring count of ca. 60 in females, some reach 182–192, and in the case of *E.persephone*, a superlative 330 rings. Even though euanamorphosis may partly explain why these millipedes have greater than average ring counts, the reason for the discontinuity in the distribution of ring numbers in the Colobognatha remains unclear (Minelli and Edgecombe 2022). So called super-elongation (> 180 rings) has evolved at least twice in the class Diplopoda, in Siphonophorida and Polyzoniida, and these millipedes drastically exceed the leg count of any animal. With significantly more than any other species of diplopod, the great number of legs in these millipedes is linked to a lifestyle burrowing through the soil (Marek et al. 2012, 2016, 2021). Adaptations, such as compressible segments due to primitively unfused sclerites, coupled with an extensible and flexible body, allows the millipede to squeeze through narrow underground crevices. The continuous metachronal wave gait and action of concentric tubular rings sliding within one another provide continuous pushing force. In combination with the many legs, longitudinal and oblique muscles pull the rings together, facilitating forward locomotion (Manton 1961). This concertina-like fashion of burrowing resembles that used by earthworms and by centipedes of the order Geophilomorpha, another group of highly elongated myriapods that includes the leggiest centipedes, *Gonibregmatusplurimipes* Chamberlin, 1920 from Fiji with 191 segments and 382 legs, and *Chomatobiusbakeri* (Chamberlin, 1912) from Los Angeles with 362 legs.

Many species of Siphonophorida await description, especially in the tropics and temperate regions where they often occur deep within the soil (Marek et al. 2016, 2021). However, their antiquated and fragmentary classification hinders taxonomic work and the primary description of species (Read and Enghoff 2018). Detailed syntheses of the group were published by Verhoeff (1940) and Attems (1951), and later revisited by Hoffman (1980), Jeekel (2001), and Shelley (1996a, 1996b). Current catalogs of the order are given in Shelley (1996b, New World Siphonophoridae), Enghoff et al. (2015; list of the genera of Siphonophorida), and Marek et al. (2016; Siphonorhinidae). Although family-level classification is primarily based on characters of the head, genera are mainly defined by male genitalia, specifically the gonopods (ninth and tenth leg pairs modified as sperm transfer organs). Although gonopodal characters appear useful for differentiation of closely related millipede species in a pairwise manner, higher-level groups inferred from them seem inaccurate (Means and Marek 2017). Furthermore, because the male gonopods of Siphonophorida are simple and leg-like (each composed of a primitive complement of seven podomeres) and are relatively conserved in morphology between species and across considerable geographical distances, some taxonomic groups that have been formed on the basis of overall similarity of gonopods may be artificial, overly inclusive, and polyphyletic. The inaccurate classificatory schemes of *Siphonophora* Brandt, 1837 and *Siphonorhinus* Pocock, 1894 exemplify this and have accumulated many species. For example, the genus *Siphonophora* encompasses ca. 80 species, mainly in the Americas, but also with species in Pakistan, Sri Lanka, Vietnam, Myanmar, Malaysia, Indonesia, Philippines, New Caledonia, New Guinea, Australia, Fiji, Solomon Islands, and New Zealand (Shelley 1996a, b; Enghoff et al. 2015). The small size of the gonopods (50 µm wide in *Illacme* spp.) and their gradual development, which can continuously change shape between stadia leading to adulthood, compounds an already challenging situation. Because there has never been a taxonomic synthesis of the Siphonophorida, including a molecular phylogenetic analysis as an independent data set to test morphological character hypotheses, an informative integrative framework for the placement of species remains unfinished (Marek and Bond 2006; Read and Enghoff 2009, 2018; Marek et al. 2012, 2016).

The definition of families by morphology of the head also may need critical reexamination. Based on a phylogeny from Marek et al. (2021), which included a sample of five species of Siphonophorida, the order is monophyletic, but the family Siphonorhinidae is paraphyletic with respect to the Siphonophoridae. These results suggest that the distinguishing characteristics of the Siphonorhinidae, which include a pear-shaped head and elbowed antennae, are shared primitive features, and the distinctive elongate beak of siphonophorids, among other distinguishing features, may have originated more recently in the diversification of the order. Specifically, Siphonorhinidae is a paraphyletic grade with respect to Siphonophoridae. Although uncovering higher level relationships of the order was not an objective of the phylogenomic analysis from Marek et al. (2021), future phylogenetic studies would benefit from an enriched taxon sampling of Siphonophorida (especially Siphonophoridae) to address questions of the systematic relationships of the group. Without informative systematic resources, and the antiquated taxonomic infrastructure of the group, progress on describing diversity in the group is hindered.

*Illacme* Cook & Loomis, 1928, the sole representative of the family Siphonorhinidae in the Western Hemisphere, is noticeably distinct from other genera in the order Siphonophorida. In combination with its super-elongated trunk, the taxon possesses distinct features that differentiate it from other Siphonophorida, such as its pear-shaped head and lack of a beak (contrasting with other siphonophoridan genera in the Western Hemisphere), elbowed antennae, and small basiconic sensilla in a shallow depression on the antennae. The genus differs from other siphonorhinids in its posterior gonopodal podomere 7 divided into 3–5 branches with one branch spike-like (Marek et al. 2016). *Illacme* is sister to the monotypic African genus *Nematozonium* (Shelley and Hoffman 2004; Marek et al. 2016, 2021). Eighty years after the description of *I.plenipes*, a second species, *Illacmetobini* Marek, Shear & Krejca, 2016, was discovered in a marble cave in Sequoia National Park, California (Marek et al. 2016). Here we describe a third species of the genus *Illacme* with 125 rings and 486 legs from the Los Angeles Basin of southern California.

## ﻿Materials and methods

### ﻿Collections

On 2 April 2018, CL and JB discovered a small white siphonophoridan millipede in Lake Forest, California, and recorded its observation in the citizen science website iNaturalist (Ueda et al. 2019). Then, PEM and CLH collected specimens on 21 December 2018, and based their field work on the geographical coordinates and metadata associated with the original iNaturalist observation. Subsequently, PEM confirmed that the millipede was an undescribed species of *Illacme* in the family Siphonorhinidae based on the diagnoses in Marek et al. (2012, 2016). We selected locations to search for millipedes based on similarity to the habitats and microhabitats of *I.plenipes* in San Benito County, California. Specifically, we examined oak woodland habitats and microhabitats of the undersides of stones and decaying oak logs (Marek et al. 2012). The stones, detritus (bark and dead leaves), and humus layer were carefully removed, and the underlying soil was gently excavated to a depth of ca. 3 cm. Removed organic material was then replaced in a similar orientation to prevent desiccation. Collecting ethics followed the Insect Collectors’ Code (Trietsch and Deans 2018). Due to the very slow locomotion of *I.plenipes*, its endogean lifestyle, and likeness in appearance to root hairs of plants, any object that was pale, narrow (< 1 mm wide) and longer than 20 mm was carefully examined by eye for more than 2 s to discern movement and identity. Due to their fragility and diminutive size, millipedes were collected by gently prying the middle of the body from beneath with a thin wooden twig and placing them into a 20-mL plastic snap cap vial with soil from their microhabitat to prevent desiccation and for cushioning in transport. In total, ca. 35 millipedes were encountered and 23 individuals were collected: 11 females, 9 males, and 3 juveniles. One female individual was collected from directly beneath a ca. 4-m long oak log (*Quercusagrifolia* Née, 1801) and the others were found beneath decaying leaves and within the matrix of ca. 3 cm of soil covered by a large pile of downed oak logs. On 18 January 2022, MCB collected ten additional specimens: four males, five females, and one sex-indeterminate juvenile. He recorded his observations on iNaturalist (iNaturalist observation: 105275476). Location data of the sites were recorded on a collection card according to Means et al. (2015). The information was included on specimen labels associated with natural history specimens and was databased in the biodiversity data management system Symbiota Collections of Arthropods Network (http://symbiota4.acis.ufl.edu/scan/portal/).

### ﻿Photography

Habitat photographs were captured with an iPhone 7 camera and millipede photographs and videos were recorded with a Canon EOS 5D Mark IV digital SLR camera and a 65 mm Canon MPE lens. To record burrowing behavior, a female millipede (MPE04624) and soil from its microhabitat were placed between two pieces of plate glass and mounted vertically in front of a Canon 5D camera on a tripod and illuminated with a GoBe 700-wide LED light (Light & Motion Inc., California). The results of video analysis of movement and locomotion are described below in the species taxonomy section under the heading of ‘Behavior’.

### ﻿Electron microscopy

We used scanning electron microscopy (SEM) to examine morphology of *I.socal* sp. nov. at 114–12662× magnification. Images were acquired using a FEI Quanta 600 FEG environmental scanning electron microscope (FEI Inc., Hillsboro, Oregon). We prepared a male and female specimen for SEM. Millipedes were live-sectioned transversely into four parts (anterior, two middle trunk sections, posterior) with a flame-sterilized straight-edge razor. Specimens were then ethanol preserved (80%), air-dried, and affixed to a 12.7-mm diameter aluminum SEM stub with double-sided carbon tape (Ted Pella Inc., California), or with graphite conductive adhesive #112 (Electron Microscopy Sciences). Stubs were then plasma-coated under stable argon pressure with a 40-nm thick layer of a mixture of palladium and platinum metals in a Leica EM ACE600 High Vacuum Coater (Leica Microsystems, Wetzlar, Germany). Coated stubs were imaged using a 3.5-spot size and at 5 kV. Micrographs were captured as 16-bit 4096 × 3775-pixel grayscale images and edited in Adobe Photoshop CC and composed as figures in Illustrator CC 5 (Adobe Inc., California). The uncompressed and uncropped scanning electron micrographs of *I.socal* sp. nov. are deposited in the Dryad Data Repository at https://doi.org/10.5061/dryad.x95x69pq7 under a public domain CC0 Creative Commons license.

### ﻿Specimen preparation

After photography and videography, specimens were preserved and deposited in the Virginia Tech Insect Collection (VTEC, https://collection.ento.vt.edu). An approximately 1-cm length of the body trunk from four live specimens (two females and two males) was preserved in 100% ethanol and archived at -80 °C for later extraction of DNA. The remaining body tissue was then preserved in 80% ethanol. Other specimens were preserved directly in 100% ethanol and stored in 8 mL screw cap vials at -20 °C. Three specimens were preserved in 100% methanol for later chemical analysis.

### ﻿DNA sequencing

From ethanol preserved tissue, genomic DNA was extracted and purified with a Qiagen DNEasy tissue kit following the manufacturer’s protocol (Qiagen, Germany). Remaining tissue and genomic DNA stored in Qiagen AE buffer were deposited in the Virginia Tech Insect Collection freezer storage (https://collection.ento.vt.edu). Genomic DNA was used as a template to amplify a fragment of the cytochrome c oxidase subunit I gene (COI) using polymerase chain reaction (PCR) and the Folmer et al. (1994) primers LCO1490 (forward) and HCO2198 (reverse) according to the procedures described in Means and Marek (2017). Amplified DNA was then cleaned, concentration quantified and normalized, and sequenced at the University of Arizona Genetics Core using an Applied Biosystems 3730 DNA Analyzer (Applied Biosystems, Foster City, CA, USA). DNA chromatograms were trimmed, bases called, and overlapping fragments made into a 612-basepair contiguous sequence in Mesquite using phred and phrap in the Chromaseq module (Ewing et al. 1998; Maddison and Maddison 2010). Genomic DNA was sequenced with whole genome sequencing using an Illumina Nextseq platform according to methods described in Marek et al. (2021). Pairwise distance was calculated by dividing the number of nucleotide differences by the total number of nucleotides compared. The diagnosis of *I.socal* sp. nov. included nucleotide site substitutions that are the unique states of its COI sequence identified in Mesquite using the ‘With State Distinguishing Selected Taxa’ tool (Maddison and Maddison 2010). The site numbers of the unique states are supplied in parentheses. The unique sites of *I.socal* sp. nov. are those nucleotides that differ from *I.plenipes* based on a pair-wise alignment. The site numbers are those from the 612 base-pair COI sequence of *I.socal* sp. nov. as a reference (U.S. National Center for Biotechnology Information accession numbers for *I.socal* sp. nov., MPE04622: COI, MT506032; WGS, SRX12626610; *I.plenipes*, SPC001187: COI, JX962724; WGS, SRX12626606). A corresponding fragment of COI for *I.tobini* was not available due to degradation of its genomic DNA (Marek et al. 2016).

### ﻿Materials examined

To compare *I.socal* sp. nov. with the two other species in the genus *Illacme*, *I.plenipes* and *I.tobini*, we examined material in natural history collections from the Smithsonian Institution (**USNM**), Florida State Collection of Arthropods (**FSCA**), Virginia Museum of Natural History (**VMNH**), and Virginia Tech Insect Collection (**VTEC**). Examination of specimens at magnifications 8–100× was accomplished with a Leica M125 stereomicroscope illuminated by a Leica LED5000 spotlight illuminator (Wetzlar, Germany). The following dimensions were measured for *I.socal* sp. nov., (1) body length: measured from anterior margin of labrum to posterior margin of paraprocts, abbreviated BL; (2) head width, HW; (3) head length, HL; (4) interantennal socket width, ISW; (5) antennomere 6 width, AW; (6) collum width, CW; (7) metazonite width at the anterior 1/4 length of body, W1; (8) metazonite length at the anterior 1/4 length of body, L1; (9) metazonite height at the anterior 1/4 length of body, H1; (10) first apodous metazonite width, AS1; (11) anterior gonopod podomere 7 width, A7W; and (12) posterior gonopod podomere 7 width, P7W. These 12 measurements refer to those in Marek et al. (2016), and 1–10, 17, and 18 are used in Marek et al. (2012). Specimen dimensions were measured from specimens using an eyepiece ocular reticule calibrated with a stage micrometer, and from digital scanning electron and light micrographs using the segmented line measurement tool in ImageJ64 (Rasband 2011). Measurements are reported in millimeters and this unit abbreviation is hereafter omitted. The number of rings were counted and legs calculated using the formula **l** = ((**p** + **a**) × 4)–(**a** × 4)–(10), where **l** is the number of legs, **p** is the number of podous rings (each bearing two leg pairs), **a** is the number of apodous rings (without legs), and 10 is the number to be subtracted because the first ring (the collum) is legless and rings 2–4 (the millipede “thorax”) each have one pair of legs (Marek et al. 2016). The identification and terminology of antennal sensilla followed that of Nguyen Duy-Jacquemin (1974) and Chung and Moon (2006), summarized and reviewed by Sombke and Ernst (2014). The terminology of mouthparts is from Silvestri (1903), Koch (2015), and Moritz et al. (2022).

### ﻿Abbreviations

Museum abbreviations are as follows: Field Museum of Natural History (**FMNH**); University of California, Davis, Bohart Insect Collection (**UCDC**); Virginia Museum of Natural History (**VMNH**); and Virginia Tech Insect Collection (**VTEC**). Supplementary abbreviations are the following: **HT** = holotype and **PT** = paratype.

## ﻿Results

### ﻿*Illacme* taxonomy

**Class Diplopoda de Blainville in Gervais, 1844**

**Subclass Chilognatha Latreille, 1802/1803**

**Infraclass Helminthomorpha Pocock, 1887**

**Subterclass Colobognatha Brandt, 1834**

**Order Siphonophorida Hoffman, 1980**

#### ﻿Family Siphonorhinidae Cook, 1895

##### ﻿Genus Illacme

Taxon classificationAnimaliaSiphonophoridaSiphonorhinidae

Cook & Loomis, 1928

76E0BB57-44BE-5C83-9314-2D0690822167

###### Type species.

*Illacmeplenipes* Cook & Loomis, 1928.

###### Species included.

*Illacmeplenipes* Cook & Loomis, 1928; *Illacmetobini* Marek, Krejca & Shear, 2016; *Illacmesocal* sp. nov.

###### Family placement.

**Figure 1.**
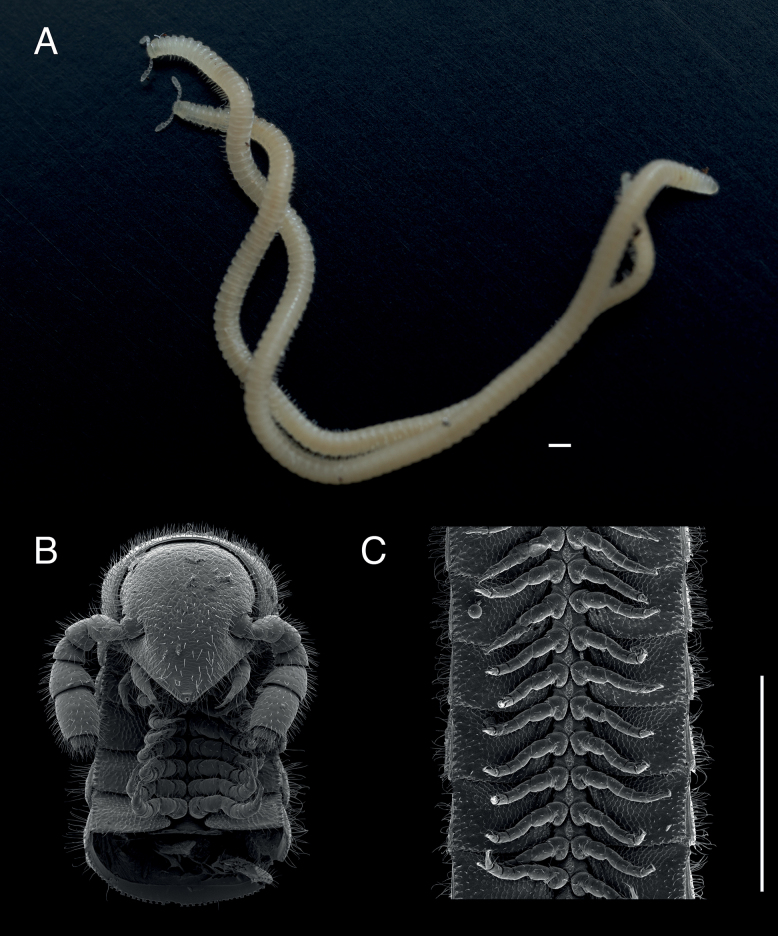
*Illacmesocal* sp. nov. **A** ♂ holotype, MPE04621, and ♀ paratype, MPE04622 (with head at top) **B** scanning electron micrograph of the head of ♀, MPE04625 **C** micrograph of midbody rings of ♀, ventral view, MPE04625. Scale bars: 1 mm (**A**); 0.5 mm (**B, C**).

**Figure 2.**
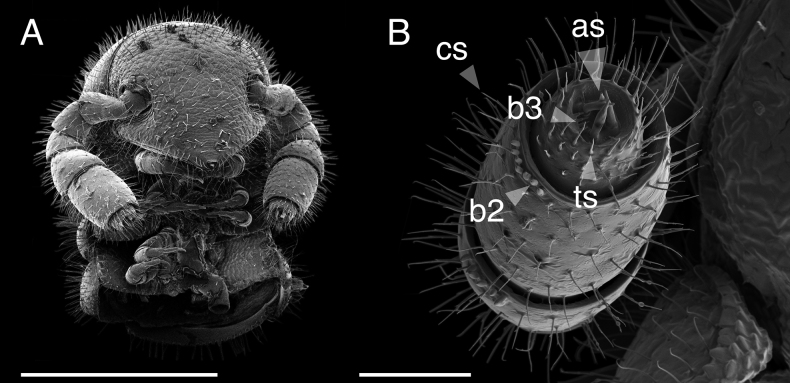
*Illacmesocal* sp. nov. Scanning electron micrographs **A** left, head and anterior rings of ♂ holotype, ventral view (MPE04621) **B** right antenna of ♀ paratype, apical view (MPE04976). Scale bars: 400 µm (**A**); 50 µm (**B**). Abbreviations: **as**, apical cones; **b2**, small basiconic sensillum; **b3**, spiniform basiconic sensillum; **cs**, chaetiform sensillum; **ts**, trichoid sensillum.

**Figure 3.**
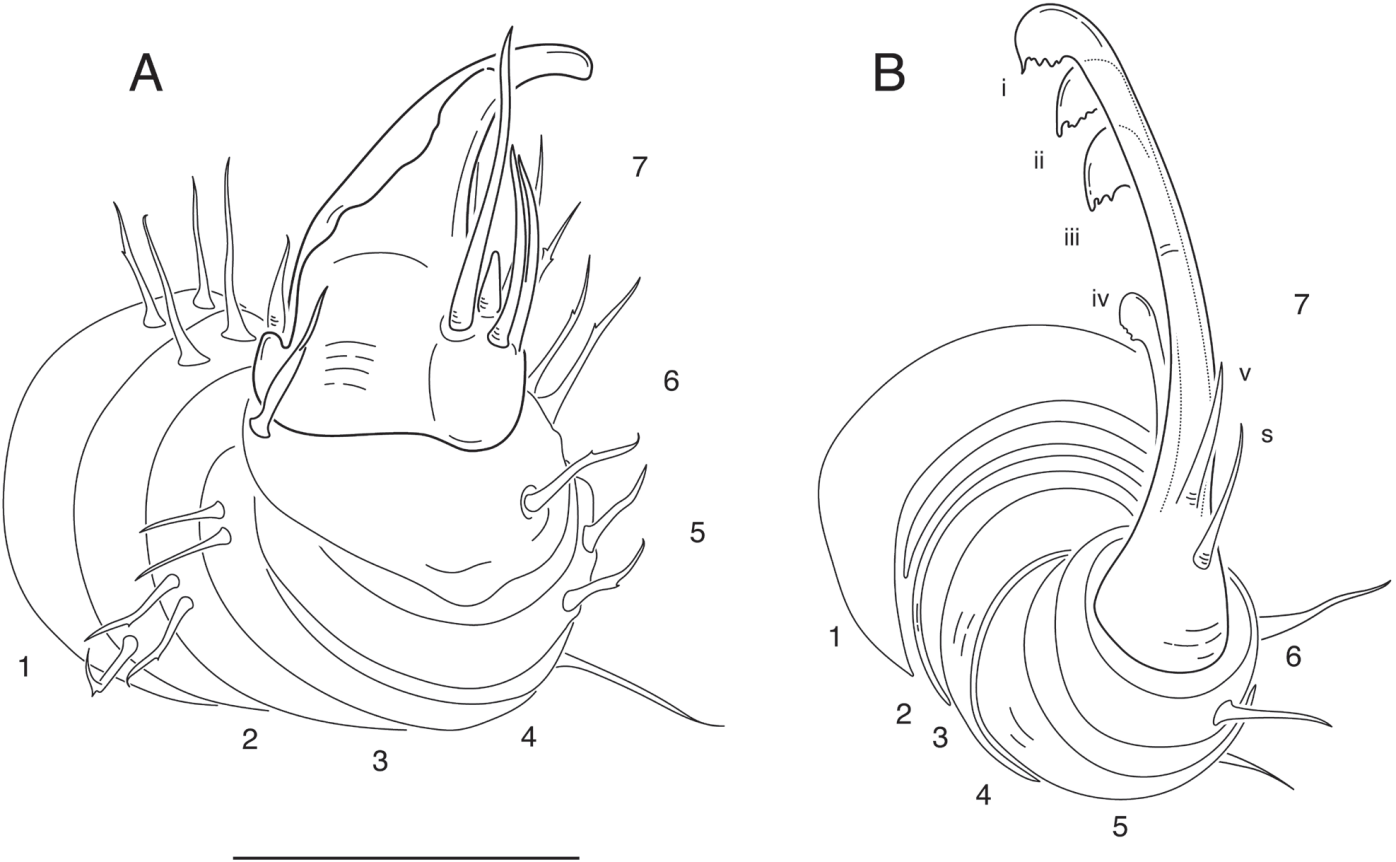
*Illacmesocal* sp. nov. ♂ **A** anterior gonopod, left side, medial view **B** posterior gonopod, left side, medial view. Podomeres numbered. Scale bar: 50 µm (**A, B**). Abbreviations: **i** – **v**, styliform articles of the posterior gonopodal apex (podomere 7); **s**, accessory seta.

The genus *Illacme* is placed in the family Siphonorhinidae based on the following morphological characters: Head pear-shaped (♂) or triangular (♀), not elongate nor with a beak, as in the Siphonophoridae (♂ Suppl. materials 1, 9: figs S1, S2, S32, S34; ♀ Fig. 1; Suppl. material 11: figs S36, S39). Antennae elbowed between antennomeres 3, 4 (Figs 1, 2; Suppl. materials 1, 10, 11: figs S2–S4, S36, S41). Antennomere 1 set deep in cranium, not fully visible dorsally as in Siphonophoridae (Suppl. material 1: figs S2, S4). Antennomere 2 longer than wide, conical, not doughnut-shaped nor wider than long as typical in Siphonophoridae (Suppl. material 1: figs S1, S4). Anterior margin of collum straight, not medially emarginate as in Siphonophoridae. Sterna with prominent midline triangular ridge, projecting ventrally (Suppl. material 5: fig. S19). Posterior gonopods with distal podomere divided into 2–5 branches with one branch spike-like (**i–v**, Fig. 3B; Suppl. material 7: figs S25–S27). See also diagnoses of *Illacme* in Shelley (1996b: 23), Marek et al. (2012: 85; 2016: 7), and Enghoff et al. (2015: 386); and of Siphonorhinidae in Shelley and Hoffman (2004: 218), Wesener (2014: 417), and Enghoff et al. (2015: 386).

##### ﻿ Illacme socal

Taxon classificationAnimaliaSiphonophoridaSiphonorhinidae

Marek & Shear sp. nov.

38DB2C32-6884-5B26-A9D7-F73380B717D6

https://zoobank.org/BE9ACD34-40AB-414F-B8D8-AF3ED0952ABA

Figs 1 , 2 , 3 , 4 ; Suppl. materials 1 , , , , , , , , , , , 12: figs S1–S42, movie S1 Vernacular name: “The Los Angeles Thread Millipede”

Illacme “Santa Ana” Marek et al. 2021: 3.

###### Type material.

***Holotype***: United States – **California** • ♂; Orange County, Lake Forest, Whiting Ranch Wilderness Park, junction of Serrano and Line Shack roads; 33.67943°N, -117.64629°W, elev. 272.8 m; 21 December 2018; 13:28; P. Marek, C. Hall leg.; VTEC, MPE04621. ***Paratypes***: United States – **California** • 8 ♂, 11 ♀; same collection data as for holotype; VTEC, MPE04622, MPE04963–4977; VMNH, MPE04624; UCDC, MPE04625. **Non-type material**: United States – **California** • 4 ♂, 5 ♀; Orange County, Lake Forest, Whiting Ranch Wilderness Park, junction of Serrano Road and Live Oak Trail; 33.679406°N, -117.647208°W, elev. 268 m; 18 January 2022; 16:41; M. Berger leg.; VTEC, MPE05265–5274.

###### Diagnosis.

Adult males of *I.socal* sp. nov. are distinct from *I.plenipes* and *I.tobini* based on the combination of: Metazonites slightly wider than prozonites, with faintly enlarged paranota (Suppl. material 5: fig. S17), not subequal in width as in *I.plenipes* nor noticeably wider as in *I.tobini*. Ozopore peritreme with two large backwards projecting spines (sp, Suppl. material 5: fig. S20) as in *I.plenipes*, not lacking two large spines as in *I.tobini*. Ozopore ringed with ca. 14 setae. Ozopores situated inside (mediad) lateral margin, oriented dorsally (Suppl. material 5: fig. S17) as in *I.plenipes*, not dorsolaterally and near lateral margin as in *I.tobini*. Metazonite posterior margin (limbus) lined with anchor-shaped, posteriorly projecting spines as in *I.plenipes* (**an**, Suppl. material 5: figs S17, S20); spines not quadrate-shaped as in *I.tobini*. Posterior margin of metazonite straight as in *I.plenipes*, not sinuate with anteriorly curved paramedial margins as in *I.tobini* (Suppl. material 5: fig. S17). Telson densely covered with irregularly oriented and unevenly distributed stout spines on lateral surface only (Suppl. material 6: fig. S22) as in *I.tobini*; telson not covered with stout spines on all surfaces nor with posterior margin lined with posterodorsally oriented anchor-shaped spikes as in *I.plenipes*. Hypoproct with > 2 setae present arranged in a setal row as in *I.plenipes* (Suppl. material 6: fig. S22), not as in *I.tobini* with two setae. Anterior gonopodal apex (podomere 7) with four spines (+ 1 tarsungulum) (Fig. 3A; Suppl. materials 6, 7: figs S23–S27), not three spines (+ 1 tarsungulum) as in *I.plenipes* nor spinose with eight spines (+ 1 tarsungulum) as in *I.tobini*. Anterior gonopodal podomere 3 with two long setae as in *I.tobini* (Fig. 3A), not ringed with six setae as in *I.plenipes*. Posterior gonopodal apex (podomere 7) comprising a bundle of five styliform articles, with one article (the tarsungulum) spike-shaped **(i–v**, Fig. 3B; Suppl. material 7: figs S25–S27), not bundle of three styliform articles as in *I.plenipes* nor four styliform articles as in *I.tobini*. The differential diagnosis of *I.socal* sp. nov., *I.tobini* and *I.plenipes* is summarized in Table 1, and a comparison of measurements between these species for a male individual with an equivalent number of rings shown in Table 2.

**Table 1.** Differential diagnostic characters of *Illacmesocal* sp. nov., *Illacmetobini* and *Illacmeplenipes*. (*) indicates revisions to Table 1 from Marek et al. 2016. (**) indicates that there is also a seta below the bundle that is not counted.

	*Illacmetobini*	*Illacmeplenipes*	*Illacmesocal* sp. nov.
**Rings**	Metazonites wider than prozonites (Marek et al. 2016: fig. 10A)	Metazonites subequal in width (Marek et al. 2016: fig. 10B)	Metazonites slightly wider than prozonites (Suppl. material 5: fig. S17)
**Peritreme**	2 large backwards projecting spines absent (Marek et al. 2016: fig. 16E)	2 large backwards projecting spines present (Marek et al. 2016: fig. 16F)	2 large backwards projecting spines present (**sp**, Suppl. material 5: fig. S20)
Metazonite anterior margin adornment*	Without tubercles or adornment along anterior margin of metazonite*	3 or 4 stout flat tubercles opposite ozopore near anterior margin, lunate arrangement encircling ozopore*	Row of stout flat tubercles along anterior margin of metazonite; tubercles absent medially
**Metazonite posterior margin adornment**	Lined with quadrate backwards projecting spines (Marek et al. 2016: fig. 10C, E)	Lined with anchor-shaped backwards projecting spines (Marek et al. 2016: fig. 10D, F)	Lined with anchor-shaped backwards projecting spines (**an**, Suppl. material 5: figs S17, S20)
**Metazonite posterior margin shape**	Sinuate, with anteriorly curved paramedial margins (Marek et al. 2016: fig. 10A)	Straight, without curvature (Marek et al. 2016: fig. 11B)	Straight, without curvature (Suppl. material 5: fig. S17)
**Telson**	Covered with stout spines on lateral surface only (Marek et al. 2016: fig. 11A)	Covered with stout spines on all surfaces (Marek et al. 2016: fig. 11B)	Covered with stout spines on lateral surface only (Suppl. material 6: fig. S22)
**Hypoproct**	2 setae present (Marek et al. 2016: fig. 11A)	> 2 setae present, in a setal row (Marek et al. 2016: fig. 11B)	> 2 setae present, in setal row (Suppl. material 6: fig. S22)
**Anterior gonopodomere 3**	2 setae present (Marek et al. 2016: fig. 8E)	6 setae present (Marek et al. 2016: fig. 8F)	2 setae present (Fig. 3A)
**Anterior gonopodal apex**	8 spines + 1 tarsungulum (Marek et al. 2016: fig. 9C)*	3 spines + 1 tarsungulum (Marek et al. 2016: fig. 9D)*	4 spines + 1 tarsungulum (Fig. 3A; Suppl. materialс 6, 7: figs S23–S27)
Posterior gonopodal apex**	Bundle of 4 styliform articles, with one these articles (the tarsungulum) spike-shaped (Marek et al. 2016: figs 11C, 12B)*	Bundle of 3 styliform articles, with one these articles (the tarsungulum) spike-shaped (Marek et al. 2016: fig. 11D)*	Bundle of 5 styliform articles, with one these articles (the tarsungulum) spike-shaped (**i**–**v**, Fig. 3B; Suppl. material 7: figs S25–S27)

**Table 2.** Comparison of counts (p, a, l, p + a + T) and measurements between *Illacmesocal* sp. nov., *Illacmetobini*, and *Illacmeplenipes* for a male individual with an equivalent number of rings. Measurements are reported in millimeters.

	p	a	l	HW	HL	ISW	AW	CW
***I.tobini* (MPE00735)**	106	2	414	0.34	0.39	0.21	0.11	0.44
***I.plenipes* (SPC000932)**	105	2	402	0.31	0.40	0.19	0.10	0.40
***I.socal* sp. nov. (MPE04621)**	102	1	398	0.31	0.39	0.20	0.10	0.40
***I.socal* sp. nov. (MPE04622)**	118	1	462	0.32	0.38	0.21	0.10	0.40
	**W1**	**L1**	**H1**	**AS1**	**A7W**	**P7W**	**BL**	**p + a + T**
***I.tobini* (MPE00735)**	0.52	0.20	0.31	0.43	0.04	0.03	19.73	106 + 2 + T
***I.plenipes* (SPC000932)**	0.40	0.16	0.40	0.43	0.05	0.04	17.12	105 + 2 + T
***I.socal* sp. nov. (MPE04621)**	0.50	0.12	0.40	0.43	0.04	0.02	18.93	102 + 1 + T
***I.socal* sp. nov. (MPE04622)**	0.52	0.18	0.39	0.38	NA	NA	22.47	118 + 1 + T

Nucleotide site substitutions. COI: A (36, 48, 51, 57, 67, 70, 75, 84, 85, 135, 138, 153, 156, 165, 181, 195, 198, 213, 243, 246, 280, 291, 294, 297, 312, 321, 366, 390, 447, 471, 486, 519, 522), C (18, 33, 55, 132, 134, 162, 172, 180, 201, 207, 240, 252, 259, 273, 276, 327, 333, 360, 361, 403, 414, 417, 429, 435, 464, 493, 505, 517, 523, 525), G (32, 74, 97, 100, 117, 120, 216, 264, 287, 292, 300, 433, 454, 501), T (25, 30, 54, 81, 96, 102, 105, 114, 147, 177, 222, 258, 262, 282, 303, 318, 369, 372, 378, 384, 393, 396, 409, 420, 423, 450, 459, 468, 469, 483, 504, 556, 559, 561, 570, 576).

###### Description of holotype.

**Figure 4.**
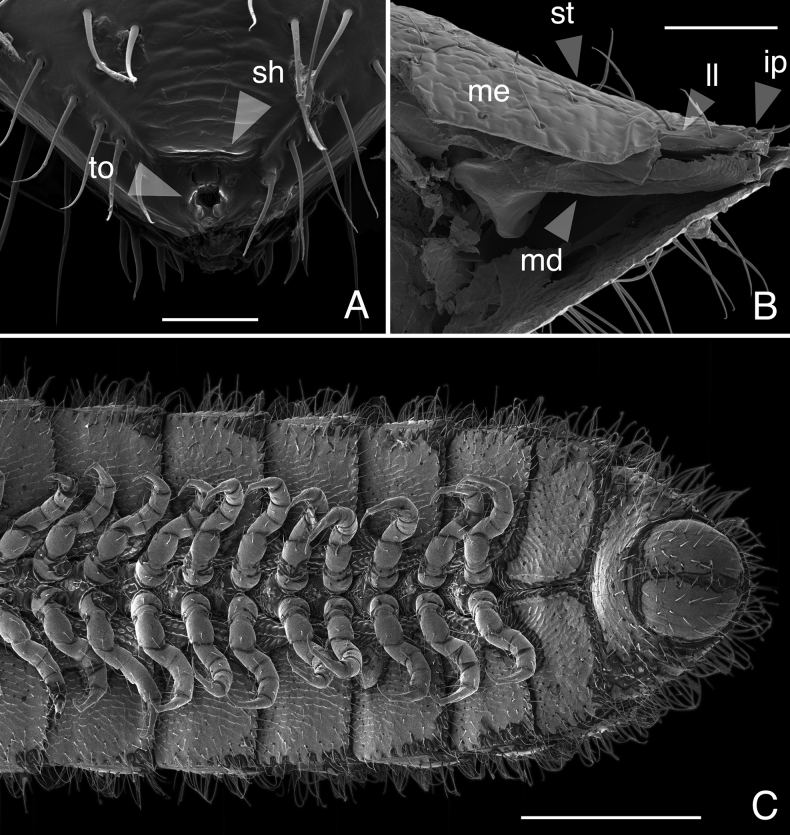
*Illacmesocal* sp. nov. Scanning electron micrographs **A** labrum of ♀ paratype, dorsal view (MPE04625) **B** mandible of ♂ paratype, lateral view (medially sectioned with left side of head removed), gnathochilarium at top (MPE04971) **C** posterior-most rings and telson of ♂ holotype, ventral view (MPE04621). Scale bars: 50 µm (**A, B**); 250 µm (**C**). Abbreviations: **ip**, inner palps; **ll**, lamellae linguales; **md**, mandible; **me**, mentum; **sh**, shelf-like carina projecting dorsally from labrum-epistome margin; **st**, stipes; **to**, anteromedial tooth-lined orifice on labrum.

(♂) (Fig. 1). Counts and measurements: p = 102. a = 1. l = 398. (102 + 1 + T). BL = 18.93. HW = 0.31. HL = 0.39. ISW = 0.20. AW = 0.10. CW = 0.40. W1 = 0.50. L1 = 0.12. H1 = 0.40. AS1 = 0.43. A7W = 0.04. P7W = 0.03. Head pear-shaped, tapered anteriorly to round point at a 130° angle from antennal sockets; occiput gradually curved medially towards occipital foramen (Suppl. materials 1, 9: figs S1, S2, S32, S34). Head covered with long slender, erect setae (Fig. 2; Suppl. materials 1, 9: figs S1–S3, S32). Gnathochilarium and labrum closely appressed, tapered anteriorly to round point (Suppl. materials 1, 3, 9: figs S1, S3, S10–S12, S32). Labrum with anteromedial tooth-lined orifice (**to**, Fig. 4; Suppl. materialS 3,4: figs S9, S13, S14). Labral surface without noticeable pores (Fig. 4). (However, the apparent lack of labral pores may be a result of specimen preparation for microscopy. Labral pores are present in *I.plenipes* and *I.tobini*. A few pores may be visible in Fig. 4A). Shelf-like carina projecting dorsally from labrum-epistome margin (**sh**, Fig. 4; Suppl. material 4: figs S13, S15). Gnathochilarium and head capsule noticeably separate, with thin gap visible between (**gp**, Suppl. material 3: fig. S10). Gnathochilarium thin plate-like, occupying nearly entire ventral surface of head (Suppl. material 3: fig. S10). Gnathochilarium tightly appressed to ventral surface of head capsule, leaving a small gap anteriorly between labrum, gnathochilarial stipes. Gnathochilarium with sclerites: stipes (**st**), mentum (**me**), postmentum (**pm**), lamellae linguales (**ll**), cardines (**cd**) (**cd**, **ll**, **me**, **pm**, **st**, Suppl. material 3: fig. S10). Gnathochilarial cardo (mistakenly homologized with the mandibular cardo in Marek et al. 2016) noticeable between head capsule, gnathochilarial stipes (**cd**, Suppl. material 3: fig. S10). Stipes of gnathochilarium with inner, outer palps; outer, inner palps with 2, 3 setae, respectively (**ip**, **op**, Fig. 4; Suppl. materials 3, 4: figs S10, S13). Lamellae linguales with subapical palps (**lp**, Suppl. material 3: fig. S10). Mandibles not externally visible (Fig. 4; Suppl. materials 1, 3: figs S1, S10). Mandible spear shaped, ca. 1/2 length of gnathochilarium (Fig. 4; Suppl. materials 3, 4: figs S11, S15). Mandible with pectinate lamella and four or five flabellate external teeth (**md**, **et**, **pl**, Suppl. material 4: figs S13–S16). Molar plate absent. Mandibular pectinate lamella with numerous rows of jagged ventrally projecting serrulae, nested in groove of endochilarial frontal body (Fig. 4; Suppl. material 4: figs S13–S16). Endochilarium with V-shaped frontal body. Endochilarium with fringed lobes (‘spatulae’ sensu Silvestri, 1903) that protrude distally through gnathochilarial stipes and lamellae linguales (**fl**, Fig. 4; Suppl. material 4: figs S13, S14). Antennae sub-geniculate, elbowed between antennomeres 3 and 4 (Figs 1, 2; Suppl. materials 1, 10, 11: figs S1–S4; S36, S42). Antennae comprising eight antennomeres, 5 and 6 enlarged. Five sensillum types: four apical cones (AS) oriented in a trapezoidal cluster on eighth antennomere, with longitudinally grooved outer surface and circular pores apically (**as**, Fig. 2; Suppl. material 2: figs S6–S8). Chaetiform sensilla (CS) widely spaced on antennomeres 1–7, each sensillum with one or two barbules (**cs**, Fig. 2; Suppl. materials 1, 2: figs S1–S8). Trichoid sensilla (TS) oriented apically encircling antennomeres 6, 7, lacking barbules (**ts**, Fig. 2; Suppl. material 2: figs S5, S6). Small basiconic sensilla (Bs_2_) in rows of six and seven oriented apical dorsally (retrolaterally) on antennomeres 5 and 6; smooth, capsule-shaped, 1/2 length of chaetiform sensillum (**b2**, Fig. 2; Suppl. material 2: figs S5, S6). Spiniform basiconic sensilla (Bs_3_) in row of 4, oriented apical dorsally on antennomere 7 (on longitudinal axis with Bs_2_ on antennomeres 5, 6); sensilla tips facing apical cones; each sensillum with ca. 3 barbules encircling tip (**b3**, Fig. 2; Suppl. material 2: figs S6, S7). Two auxiliary spiniform basiconic sensilla, each on distal rim of antennomere 7 oriented 120° from row of four (Suppl. material 2: fig. S6). Antennae extend posteriorly to middle of tergite 3. Relative antennomere lengths 6>2>5>3>4>1>7>8. Collum not concealing head, with straight anteromedial edge, gradually tapering laterally (Suppl. materials 1, 11: figs S1, S40–S42). Lateral margin of collum rounded, with thickened scaly carina (Suppl. materials 1, 3: figs S4, S10). This carina repeated serially on lateral tergal and pleural margins (absent from telson). Lateral tergal and pleural carinae jagged, saw-like (potentially interlocking), pronounced on midbody rings (Fig. 1, Suppl. material 5: fig. S18). Metazonites slightly wider than prozonites, with faintly enlarged paranota (Suppl. materials 5, 11: figs S17, S39, S40). Metazonites slightly arched (Suppl. material 9: fig. S32). Metazonite dorsally covered with long, slender setae (Fig. 1; Suppl. material 11: figs S40, S41). Tergal setae hollow; tipped with translucent silk-like exudate, exudate sometimes tangled with neighboring setae (**ex**, Suppl. material 9: fig. S35). Metazonite posterior margin (limbus) lined with anchor-shaped posteriorly projecting spines (**an**, Suppl. material 5: figs S17, S20). With row of conical spines anterior to limbus on ozoporiferous rings only (Suppl. material 5: figs S17, S20). Limbal anchor-shaped spikes alternating in size (large, small—sometimes large, small, small, large) along margin. Ozopores oriented dorsally, located near limbus (Suppl. material 5: fig. S17). Ozopores absent from collum, tergites 2–4, telson. Ozopores elevated faintly on peritremata (porosteles absent), with two large backwards projecting spines, encircled with ca. 14 robust setae (**sp**, Suppl. material 5: fig. S20). Row of stout flat tubercles along anterior margin of metazonite; tubercles absent medially. Posterior half of body with rings more convex (Suppl. material 10: fig. S37), posterior-most tergites covered with a greater density of long, slender setae (Suppl. material 5: fig. S18). Apodous ring without visible sternum, pleurites contiguous in midline. Apodous tergite densely setose, with unevenly distributed spikes localized to posterolateral corner; posterior margin lined with posteriorly oriented anchor-shaped spikes (Fig. 3A; Suppl. material 6: fig. S22). Telson covered with dorsally oriented stout spines on lateral surface only; without anchor-shaped spikes on margin (Suppl. material 6: fig. S22). Prozonite highly sculptured, with 2–4 rows of discoidal flat tubercles (Suppl. material 5: fig. S20). Tubercles in two shape classes: posterior prozonal tubercles button-shaped protuberant (**tb**); anterior tubercles flush (**tf**) with surface anteriorly (**tb**, **tf**, Suppl. material 5: fig. S20). Pleurites quadrate, flat, with jagged scaly lateral, posterior, medial margins (Fig. 1). Pleurite medial margin broad, with scaly raised carina (Fig. 1). Pleurites plate-like, large, composing 4/5 of ventral ring area. Pleural medial margins overlapping lateral sternal margins, covering spiracles (Fig. 1). Sternites heart-shaped, wider anteriorly. Anterior, posterior sternites free, separate from pleurites (Suppl. material 5: fig. S19). Sternum with prominent midline triangular ridge projecting ventrally, tapering to a point anteriorly (**rd**, Suppl. material 5: fig. S19). Sternum with spiracles and legs oriented posteroventrally (Fig. 1; Suppl. material 5: fig. S19). Spiracles circular, orifice open, hollow; oriented (above) dorsal to legs (Suppl. material 5: fig. S19). Tergites, pleurites, sternites separated by arthrodial membrane (Suppl. materials 5, 6: figs S18, S19, S22). Arthrodial membrane between tergites, pleurites wider posteriorly, pleated, thereby allowing telescoping body rings. Telson, paraprocts covered with long slender erect setae (Suppl. materials 5, 6: figs S18, S19, S22). Setae on epiproct margin have inflated bases (potentially glandular in nature). Paraprocts quarter-spherical, anterior margins faintly scaly (Suppl. material 6: fig. S22). Hypoproct small, ca. 1/8 area of paraproct, with four posterior projecting setae. Legs (postgonopodal) with seven podomeres (relative lengths denoted by numbers; 1 longest, 7 shortest): coxa (6), trochanter (7), prefemur (2), femur (3), postfemur (5), tibia (4), and tarsus (1). Legs with sparse setae, appearance similar to chaetiform sensilla with one or two barbules. Coxae nearly contiguous medially, separated by narrow sternal ridge. Coxa (postgonopodal legs) with large posteroventrally oriented D-shaped opening for eversible sac (Fig. 1; **es**, Suppl. material 5: fig. S19). Eversible sacs membranous, distended slightly from aperture (Fig. 1). Tarsus with pincer-like claw, dorsal claw arcuate; ventral accessory claw thinner, arcuate, 1/2 length of dorsal claw (Suppl. materials 5, 6: figs S18, S22). Second leg pair with posteriorly oriented coxal gonapophyses; rounded, protuberant. Ninth, tenth leg pairs modified into gonopods, each comprising seven podomeres (Fig. 3; Suppl. materials 6, 7: figs S23–S27). Anterior gonopod, ninth leg pair, robust, thicker than posterior gonopod, tenth leg pair (Fig. 3A; Suppl. material 7: fig. S25). Anterior gonopodal apex (podomere 7) spade-shaped; in repose cupped around flagelliform posterior gonopodal apex (podomere 7). Anterior gonopodal podomere 7 with four spines + 1 tarsungulum (Fig. 3A; Suppl. materials 6, 7: figs S23–S27). Anterior gonopodal podomere 3 with two setae. Posterior gonopodal podomere 7 deeply divided, comprising a bundle of five styliform articles, with one these articles (the tarsungulum) spike-shaped (**i**–**v**, Fig. 3A; Suppl. material 7: figs S25–S27). Four dorsal-most, longest articles laminate distally, recurving laterally, with denticulate posterior margins (**i**–**iv**, Fig. 3B; Suppl. material 7: figs S25–S27). Ventral-most, fifth article spike-like (**v**, Fig. 3B). Accessory seta located proximal to fifth spike-like article at base of podomere (**s**, Fig. 3B). Triangle-shaped sterna present between left and right gonopods of ninth and tenth leg pairs, thicker between posterior gonopods. Supplementary micrographs of *I.socal* sp. nov. are archived in the Dryad Data Repository at: https://doi.org/10.5061/dryad.x95x69pq7.

###### Description of largest paratype.

(♀) VTEC (MPE04622) – Counts and measurements: p = 118. a = 1. l = 462. (118 + 1 + T). HW = 0.32. HL = 0.38. ISW = 0.21. AW = 0.10. CW = 0.40. W1 = 0.52. L1 = 0.18. H1 = 0.39. AS1 = 0.38. BL = 22.47. Morphology similar to male holotype. In combination with its counts and measurements, the following structures of female paratype differ from male holotype. Head chevron-shaped, tapered anteriorly to round point at a 120° angle anterior from antennal sockets (Fig. 1; Suppl. material 11: figs S36, S39); occipital area posterior from antennal sockets nearly straight, faintly curved medially towards occipital foramen (Suppl. material 11: fig. S41). Cyphopods large, area 1/6 the ring area in its widest cross-section; almond-shaped, bivalvular, narrow apex oriented ventrally.

###### Variation.

There is negligible variation in coloration between live specimens. Female specimens are generally larger in size (greater head, ring width) and have more rings and legs. The predominant source of variation between specimens is ring and leg counts (Tables 2, 3). Females have a maximum of 125 rings (486 legs maximum) with a median of 94, and males a maximum of 104 rings (402 legs maximum) with a median of 73. The rings of *I.socal* sp. nov. (males and females) are uniform in length, width, and height along the trunk, but are slightly taller and more convex in posterior rings.

**Table 3.** *Illacmesocal* sp. nov. ring (p + a) and leg counts. Sorted by sex, ring count (descending).

Specimen	Sex	Ring count	Leg count
MPE05266	F	125	486
MPE04622	F	119	462
MPE05265	F	116	450
MPE04625	F	115	446
MPE04624	F	103	398
MPE04966	F	102	394
MPE04623	F	98	378
MPE04963	F	96	370
MPE05268	F	92	354
MPE05267	F	90	346
MPE04965	F	85	326
MPE05270	F	84	322
MPE04974	F	67	254
MPE04976	F	57	214
MPE04973	F	47	174
MPE04964	F	44	162
MPE04977	M	104	402
MPE04621	M	103	398
MPE04970	M	96	370
MPE05274	M	83	318
MPE05271	M	75	286
MPE05272	M	75	286
MPE04969	M	71	270
MPE05269	M	70	266
MPE04967	M	60	226
MPE04968	M	53	198
MPE04972	M	52	194
MPE04975	M	51	190

###### Ecology.

**Figure 5.**
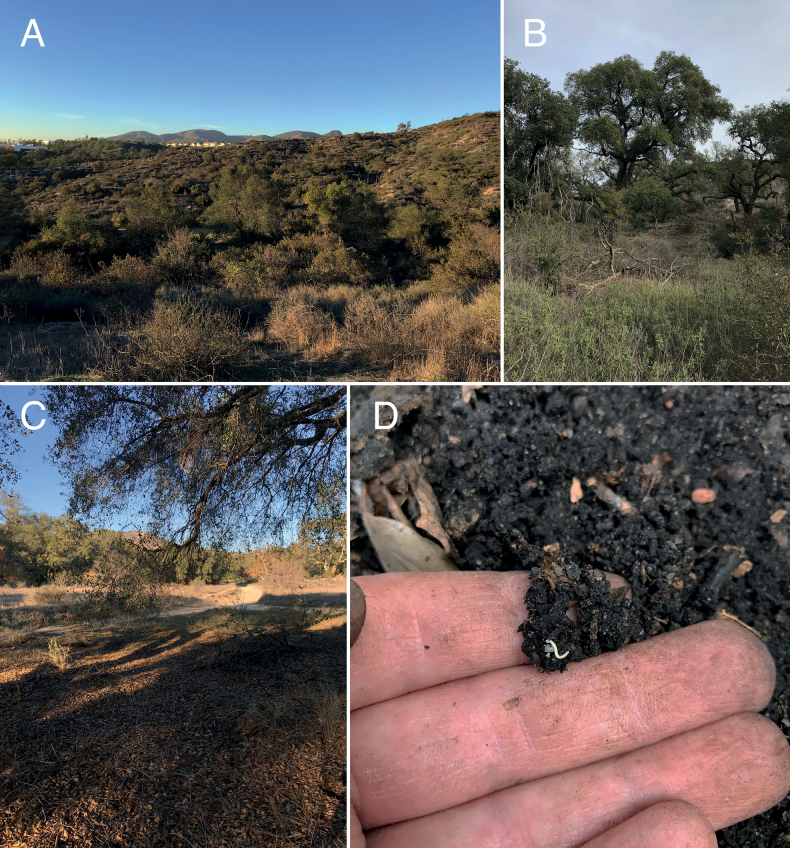
Habitat of *Illacmesocal* sp. nov. Whiting Ranch Wilderness Park, Orange County, California **A** California live oak woodland habitat surrounded by chaparral shrubland **B** close up of oak woodland habitat **C** type locality beneath oak canopy **D**an*I.socal* sp. nov. individual (center) encountered beneath the humus layer and embedded within the underlying soil matrix.

*Illacmesocal* sp. nov. individuals were encountered during the day in a California live oak woodland habitat surrounded by chaparral shrubland (Fig. 5). One female individual was found beneath a dead oak log, and the others were encountered beneath the humus layer and embedded within the underlying soil matrix (Suppl. material 8: figs S28–S31). Co-occurring dominant flora included California live-oak (*Quercusagrifolia*), California sagebrush (*Artemisiacalifornica*), California broom (*Acmisponglaber*), hollyleaf redberry (*Rhamnusilicifolia*), Pacific poison-oak (*Toxicodendrondiversilobum*), and Coastal cholla (*Cylindropuntiaprolifera*). Other invertebrates encountered included centipedes (*Arenophilusiugans* Chamberlin, 1944, *Taiyunaisantus* (Chamberlin, 1909), *Oabius* Chamberlin, 1916), beetles (*Eleodesosculans*, *Apsena* sp.), and arachnids (*Hubbardia* sp., *Cicurina* sp.). Between the collection of the type material by PEM in 2018 and nontype material by MCB in 2022, the Silverado fire (26 October–7 November 2020) burned ca. 12,500 acres including portions of Whiting Ranch Wilderness Park. The impact of this fire on the *I.socal* sp. nov. population remains unclear, but given their efficient burrowing locomotion (see behavior below), this species likely minimizes fire risk by staying in deeper soils with higher soil moisture.

###### Behavior.

When uncovered, individuals were observed spiraling downward into the soil cavity via a corkscrew-like pattern. Filmed in the laboratory, and within the soil from its microhabitat, the burrowing locomotion of a female *I.socal* sp. nov. was slow (100 µm/s), undulatory, and continuous (MPE04624, Suppl. material 12: https://vimeo.com/823446011?share=copy). The sinuous locomotory movement of the millipede appeared to follow a path of low resistance and track the topography within the soil matrix. While burrowing, a single orientation was not continually maintained, and the individual repeatedly turned and continued motion several times in different planes. While passing through a narrow junction, *I.socal* sp. nov. appeared to squeeze through the crevice by reduction of its body height by ca. 1/2 (Suppl. material 12: https://vimeo.com/823446011?share=copy, at 34 s). After squeezing into the interstice, and through cephalic nodding and dorso-ventral arching of the trunk, the millipede forcibly enlarged the interstice. The body rings did not appear to telescope into one another while passing through narrow junctions. Moving within the soil matrix, the millipede appeared to be positively thigmotactic to contact with the soil. The millipede appeared to navigate by aid of its large antennae, and detection of interstitial voids seemed to be mediated by these appendages. The antennae moved rapidly, and the left and right antennae, each equally and continually, tapped on the soil grains. As it walked, the millipede was observed asymmetrically extending one antenna into the soil lacunae ahead as if the sole appendage was used as a probe by the blind millipede to survey ahead of itself in the confined subterranean space (Suppl. material 12: https://vimeo.com/823446011?share=copy, at 30 s).

###### Distribution.

Only known from its type locality at Whiting Ranch Wilderness Park. An observation by CL from Los Angeles County at Eaton Canyon Natural Area in Pasadena, California, appears consistent with *I.socal* sp. nov. in morphological features. This millipede, a juvenile and of uncertain species identity due to lack of species-diagnostic gonopods, was observed on 10 February 2021; iNaturalist, observation: 69384055.

###### Etymology.

The species name refers to its type locality in Southern California, commonly shortened to SoCal.

## ﻿Discussion

Our discovery of the millipede *Illacmesocal* sp. nov. (family Siphonorhinidae) from the Los Angeles metropolitan area adds a third species the genus. Two species of *Illacme* are known from the Coast Ranges (*I.socal* sp. nov. and *I.plenipes*) and one is from the Sierra Nevada Range (*I.tobini*). Although members of the genus are similar in morphological features to one another, *I.socal* sp. nov. is 19.7% different in the cytochrome c oxidase subunit I (COI) barcode region to *I.plenipes* from San Benito County (California); 2–3% pairwise distance is the often-cited threshold for metazoan species differentiation (Hebert et al. 2003). The largest female of *I.socal* sp. nov. has 125 rings and 486 legs, fewer than the 192 rings and 750 legs of the largest *I.plenipes* female (Cook and Loomis 1928). The many legs and the elongated body of *I.socal* sp. nov. have been associated to its subterranean soil microhabitat and burrowing behavior (Marek et al. 2012, 2021). Through video analysis of the locomotion of *I.socal* sp. nov. in a soil matrix, we found a suite of behaviors and features that appear to facilitate endogean locomotion. These include (1) a sinuous and undulatory 3-dimensional plane of movement; (2) apparent preferential locomotion through preexisting interstitial voids largely mediated by antennation; (3) compressible rings; (4) flexible trunk, achieved through both extensibility and lateral elasticity; and (4) interstice enlargement via cephalic nodding and dorso-ventral arching of the trunk. Manton (1961) analyzed the musculature and locomotion of several members of the Siphonophorida including *Siphonophoraportoricensis* Brandt, 1837 and *Siphonophorahartii* (Pocock, 1895), species which have similar trunk architecture to *I.socal* sp. nov. with free tergites, sternites and pleurites. She highlighted the species’ dorso-ventral and lateral flexibility, spiraling locomotion, and cephalic nodding behavior, and by careful anatomical dissections, inferred what skeletomuscular features are responsible for these movements (Manton 1961). This is among the first videos analyzed of a siphonophoridan millipede burrowing and moving through small spaces and crevices underground. The locomotory behaviors of *I.socal* sp. nov. demonstrates the ease with which highly elongated millipedes travel underground, and helps explain the discovery of the 1306-legged *E.persephone* 60 m below ground. The discovery of *I.socal* sp. nov. in the Los Angeles Basin, a region with a human population of more than 18 million, shows that future studies of underground fauna, even in well-known locations, can lead to new discoveries of undescribed animal life.

Fortunately, the known occurrences of *I.socal* sp. nov., which includes the juvenile specimen from Eaton Canyon, are from two parks, which provide some protection from future development. Whiting Ranch Wilderness Park is managed by OCParks and consists of 2,500 acres of riparian and oak woodland canyons, rolling grassland hills and steep slopes of coastal sage scrub and chaparral. This park is open to hikers, mountain bikers, and equestrians. Eaton Canyon Natural Area is 198 acres and is managed by Los Angeles County Parks and Recreation. Eaton Canyon is open to hiking and equestrian use. In order to ensure the viability of *I.socal* sp. nov. and other subterranean fauna, park managers should still prioritize active conservation measures by focusing on soil preservation. Activities that disturb the soil in any way should be strongly discouraged as such activities could extirpate these populations. Further, both of the known localities are within a few meters of existing trails; therefore, visitors should be restricted from venturing off trail.

It is highly likely that *I.socal* sp. nov. also occurs in nearby private land holdings as many of these areas contain suitable habitat. Unfortunately, the relentless development pressure that has overwhelmed the greater Los Angeles area for decades will continue into the foreseeable future, putting these areas at high risk for habitat degradation and destruction. Fires, including the 2020 Silverado fire at Whiting Ranch Wilderness Park, also represent an imminent threat to soil fauna as these conserved areas contain abundant fire fuels.

## Supplementary Material

XML Treatment for Illacme

XML Treatment for Illacme socal

## ﻿Additional information

### Author ORCIDs

Paul E. Marek https://orcid.org/0000-0002-7048-2514

James Bailey https://orcid.org/0000-0001-5861-939X

William Shear https://orcid.org/0000-0002-5887-7003

### Data availability

All of the data that support the findings of this study are available in the main text or Supplementary Information.

## Supplementary materials

Supplementary material 1 *Illacmesocal* sp. nov. Scanning electron micrographs S1–S4 Data type figures (JPG images) Explanation note **fig. S1.** head, anterior rings of ♂ holotype, lateral view (MPE04621). **fig. S2.***ibid*. ♂ holotype, dorsal view (MPE04621). **fig. S3.** left antenna of ♂ holotype, anterior view (MPE04621). **fig. S4.** right antenna of ♀ paratype, dorsal view (MPE04622). Scale bars: 300 µm (**S1, S2**); 200 µm (**S3**); 100 µm (**S4**). https://binary.pensoft.net/file/866984This dataset is made available under the Open Database License (http://opendatacommons.org/licenses/odbl/1.0/). The Open Database License (ODbL) is a license agreement intended to allow users to freely share, modify, and use this Dataset while maintaining this same freedom for others, provided that the original source and author(s) are credited.Paul E. Marek, Charity L. Hall, Cedric Lee, James Bailey, Matt C. Berger, Matt T. Kasson, William Shear

Supplementary material 2 *Illacmesocal* sp. nov. Scanning electron micrographs S5–S8 Data type figures (JPG images) Explanation note **fig. S5.** left antenna of ♂ holotype, anterior view (MPE04621). **fig. S6.** right antenna of ♀ paratype, apical view (MPE04976). **fig. S7.***ibid*. ♀ paratype, apical view (MPE04622). **fig. S8.***ibid*. ♀ paratype, apical view (MPE04622). Abbreviations: **as**, apical cones; **b2**, small basiconic sensillum; **b3**, spiniform basiconic sensillum; **cs**, chaetiform sensillum; **ts**, trichoid sensillum. Scale bars: 100 µm (**S5**); 50 µm (**S6, S7**); 20 µm (**S8**). https://binary.pensoft.net/file/866985This dataset is made available under the Open Database License (http://opendatacommons.org/licenses/odbl/1.0/). The Open Database License (ODbL) is a license agreement intended to allow users to freely share, modify, and use this Dataset while maintaining this same freedom for others, provided that the original source and author(s) are credited.Paul E. Marek, Charity L. Hall, Cedric Lee, James Bailey, Matt C. Berger, Matt T. Kasson, William Shear

Supplementary material 3 *Illacmesocal* sp. nov. Scanning electron micrographs S9–S12 Data type figures (JPG images) Explanation note **fig. S9.** labrum of ♀ paratype, dorsal view (MPE04625). **fig. S10.** gnathochilarium of ♀ paratype, ventral view (MPE04976). **fig. S11.** mandible of ♂ paratype, lateral view (medially sectioned with left side of head removed), gnathochilarium at top (MPE04971). **fig. S12.***ibid*. ♂ paratype, lateral view, gnathochilarium at top (MPE04971). Scale bars: 50 µm (**S9, S12**); 200 µm (**S10**); 100 µm (**S11**). Abbreviations: **cd**, cardo; **ip**, inner palps; **ll**, lamellae linguales; **lp**, palps of lamellae linguales; **md**, mandible; me, mentum; **op**, outer palps; **pm**, postmentum; **st**, stipes; **to**, anteromedial tooth-lined orifice on labrum. https://binary.pensoft.net/file/866986This dataset is made available under the Open Database License (http://opendatacommons.org/licenses/odbl/1.0/). The Open Database License (ODbL) is a license agreement intended to allow users to freely share, modify, and use this Dataset while maintaining this same freedom for others, provided that the original source and author(s) are credited.Paul E. Marek, Charity L. Hall, Cedric Lee, James Bailey, Matt C. Berger, Matt T. Kasson, William Shear

Supplementary material 4 *Illacmesocal* sp. nov. Scanning electron micrographs S13–S16 Data type figures (JPG images) Explanation note **fig. S13.** medial cross section of head of ♂ paratype, apical view with gnathochilarium at top (MPE04971). **fig. S14.***ibid*. ♂ paratype, apical view (MPE04971). **fig. S15.** medial cross section of head of ♂ paratype, apical view with labrum at top (MPE04971). **fig. S16.***ibid*., apical view ♂ paratype (MPE04971). Scale bars: 20 µm (**S13**); 10 µm (**S14**); 100 µm (**S15**); 40 µm (**S16**). Abbreviations: **et**, external teeth; **fl**, fringed lob (spatula); **md**, mandible; **pl**, pectinate lamella; **sh**, shelf-like carina projecting dorsally from labrum-epistome margin; **to**, anteromedial tooth-lined orifice on labrum. https://binary.pensoft.net/file/866987This dataset is made available under the Open Database License (http://opendatacommons.org/licenses/odbl/1.0/). The Open Database License (ODbL) is a license agreement intended to allow users to freely share, modify, and use this Dataset while maintaining this same freedom for others, provided that the original source and author(s) are credited.Paul E. Marek, Charity L. Hall, Cedric Lee, James Bailey, Matt C. Berger, Matt T. Kasson, William Shear

Supplementary material 5 *Illacmesocal* sp. nov. Scanning electron micrographs S17–S20 Data type figures (JPG images) Explanation note **fig. S17.** mid trunk rings of ♀ paratype, dorsal view (MPE04625). **fig. S18.** posterior most rings and telson of ♂ holotype, left lateral view (MPE04621). **fig. S19.***ibid*. ♀ paratype, dorsal view (MPE04625). **fig. S20.** ozopore of ♀ paratype, left lateral view (MPE04622). Scale bars: 500 µm (**S17–S19**); 50 µm (**S20**). Abbreviations: **an**, anchor-shaped, posteriorly projecting spines; **es**, eversible sac; **rd**, sternal midline triangular ridge; **sp**, 2 large backwards projecting spines; **tb**, protuberant button-shaped tubercles; **tf**, flush tubercles. https://binary.pensoft.net/file/866988This dataset is made available under the Open Database License (http://opendatacommons.org/licenses/odbl/1.0/). The Open Database License (ODbL) is a license agreement intended to allow users to freely share, modify, and use this Dataset while maintaining this same freedom for others, provided that the original source and author(s) are credited.Paul E. Marek, Charity L. Hall, Cedric Lee, James Bailey, Matt C. Berger, Matt T. Kasson, William Shear

Supplementary material 6 *Illacmesocal* sp. nov. Scanning electron micrographs S21–S24 Data type figures (JPG images) Explanation note **fig. S21.** posterior most rings and telson of ♂ holotype, ventral view (MPE04621). **fig. S22.**ibid. ♀ paratype, oblique view (MPE04622). **fig. S23.** gonopods (9^th^ and 10^th^ leg pairs) of ♂ holotype, ventral view (MPE04621). **fig. S24.***ibid*. ♂ holotype (MPE04621). Scale bars: 300 µm (**S21, S22**); 100 µm (**S23**); 50 µm (**S24**). https://binary.pensoft.net/file/866989This dataset is made available under the Open Database License (http://opendatacommons.org/licenses/odbl/1.0/). The Open Database License (ODbL) is a license agreement intended to allow users to freely share, modify, and use this Dataset while maintaining this same freedom for others, provided that the original source and author(s) are credited.Paul E. Marek, Charity L. Hall, Cedric Lee, James Bailey, Matt C. Berger, Matt T. Kasson, William Shear

Supplementary material 7 *Illacmesocal* sp. nov. Scanning electron micrographs S25–S27 Data type figures (JPG images) Explanation note **fig. S25.** gonopods (9^th^ and 10^th^ leg pairs) of ♂ holotype, ventral view (MPE04621), ♂ holotype (MPE04621). **fig. S26.***ibid*. ♂ holotype (MPE04621). **fig. S27.***ibid*. ♂ holotype (MPE04621). Scale bars: 50 µm (**S25–S27**). Abbreviations: **i–v**, styliform articles of the posterior gonopodal apex (podomere 7). https://binary.pensoft.net/file/866990This dataset is made available under the Open Database License (http://opendatacommons.org/licenses/odbl/1.0/). The Open Database License (ODbL) is a license agreement intended to allow users to freely share, modify, and use this Dataset while maintaining this same freedom for others, provided that the original source and author(s) are credited.Paul E. Marek, Charity L. Hall, Cedric Lee, James Bailey, Matt C. Berger, Matt T. Kasson, William Shear

Supplementary material 8 Habitat of *Illacmesocal* sp. nov. Whiting Ranch Wilderness Park, Orange County, California Data type figures (JPG images) Explanation note **fig. S28**, fallen oak at type locality from under which specimens were encountered from under dead leaves and in soil. **fig. S29**, close up soil microhabitat with CLH for scale. **fig. S30**, close up of soil grain structure. **fig. S31**, an*I.socal* individual (MPE04622) encountered beneath an oak log and embedded within the humus layer. https://binary.pensoft.net/file/866991This dataset is made available under the Open Database License (http://opendatacommons.org/licenses/odbl/1.0/). The Open Database License (ODbL) is a license agreement intended to allow users to freely share, modify, and use this Dataset while maintaining this same freedom for others, provided that the original source and author(s) are credited.Paul E. Marek, Charity L. Hall, Cedric Lee, James Bailey, Matt C. Berger, Matt T. Kasson, William Shear

Supplementary material 9 *Illacmesocal* sp. nov. live habitus photographs S32–S35 Data type figures (JPG images) Explanation note **figs S32–S35** ♂ holotype (MPE04621). (Note: moss species not indigenous to its habitat). Abbreviation: **ex**, translucent silk-like exudate. https://binary.pensoft.net/file/866992This dataset is made available under the Open Database License (http://opendatacommons.org/licenses/odbl/1.0/). The Open Database License (ODbL) is a license agreement intended to allow users to freely share, modify, and use this Dataset while maintaining this same freedom for others, provided that the original source and author(s) are credited.Paul E. Marek, Charity L. Hall, Cedric Lee, James Bailey, Matt C. Berger, Matt T. Kasson, William Shear

Supplementary material 10 *Illacmesocal* sp. nov. live habitus photographs S36–S38 Data type figures (JPG images) Explanation note **fig. S36**, ♂ holotype (MPE04621, bottom) and ♀ paratype (MPE04622, top). **figs S37, S38**, ♀ paratype (MPE04622). (Note: moss species not indigenous to its habitat.). https://binary.pensoft.net/file/866993This dataset is made available under the Open Database License (http://opendatacommons.org/licenses/odbl/1.0/). The Open Database License (ODbL) is a license agreement intended to allow users to freely share, modify, and use this Dataset while maintaining this same freedom for others, provided that the original source and author(s) are credited.Paul E. Marek, Charity L. Hall, Cedric Lee, James Bailey, Matt C. Berger, Matt T. Kasson, William Shear

Supplementary material 11 *Illacmesocal* sp. nov. live habitus photographs S39–S42 Data type figures (JPG images) Explanation note ♀ paratype (MPE04625). https://binary.pensoft.net/file/866994This dataset is made available under the Open Database License (http://opendatacommons.org/licenses/odbl/1.0/). The Open Database License (ODbL) is a license agreement intended to allow users to freely share, modify, and use this Dataset while maintaining this same freedom for others, provided that the original source and author(s) are credited.Paul E. Marek, Charity L. Hall, Cedric Lee, James Bailey, Matt C. Berger, Matt T. Kasson, William Shear

Supplementary material 12 Movie of a female *Illacmesocal* sp. nov Data type video file Explanation note ♀ paratype (MPE04624) burrowing within the soil from its microhabitat. https://vimeo.com/823446011?share=copy (high resolution version deposited in the Dryad Data Repository at https://doi.org/10.5061/dryad.x95x69pq7). https://binary.pensoft.net/file/866982This dataset is made available under the Open Database License (http://opendatacommons.org/licenses/odbl/1.0/). The Open Database License (ODbL) is a license agreement intended to allow users to freely share, modify, and use this Dataset while maintaining this same freedom for others, provided that the original source and author(s) are credited.Paul E. Marek, Charity L. Hall, Cedric Lee, James Bailey, Matt C. Berger, Matt T. Kasson, William Shear
