# Supplementary figures and images for: ﻿A new species of Illacme from southern California (Siphonophorida, Siphonorhinidae)
Source: Zookeys. 2023 Jun 21;1167:265–91. doi: 10.3897/zookeys.1167.102537 (PMC10308430; doi:10.3897/zookeys.1167.102537)

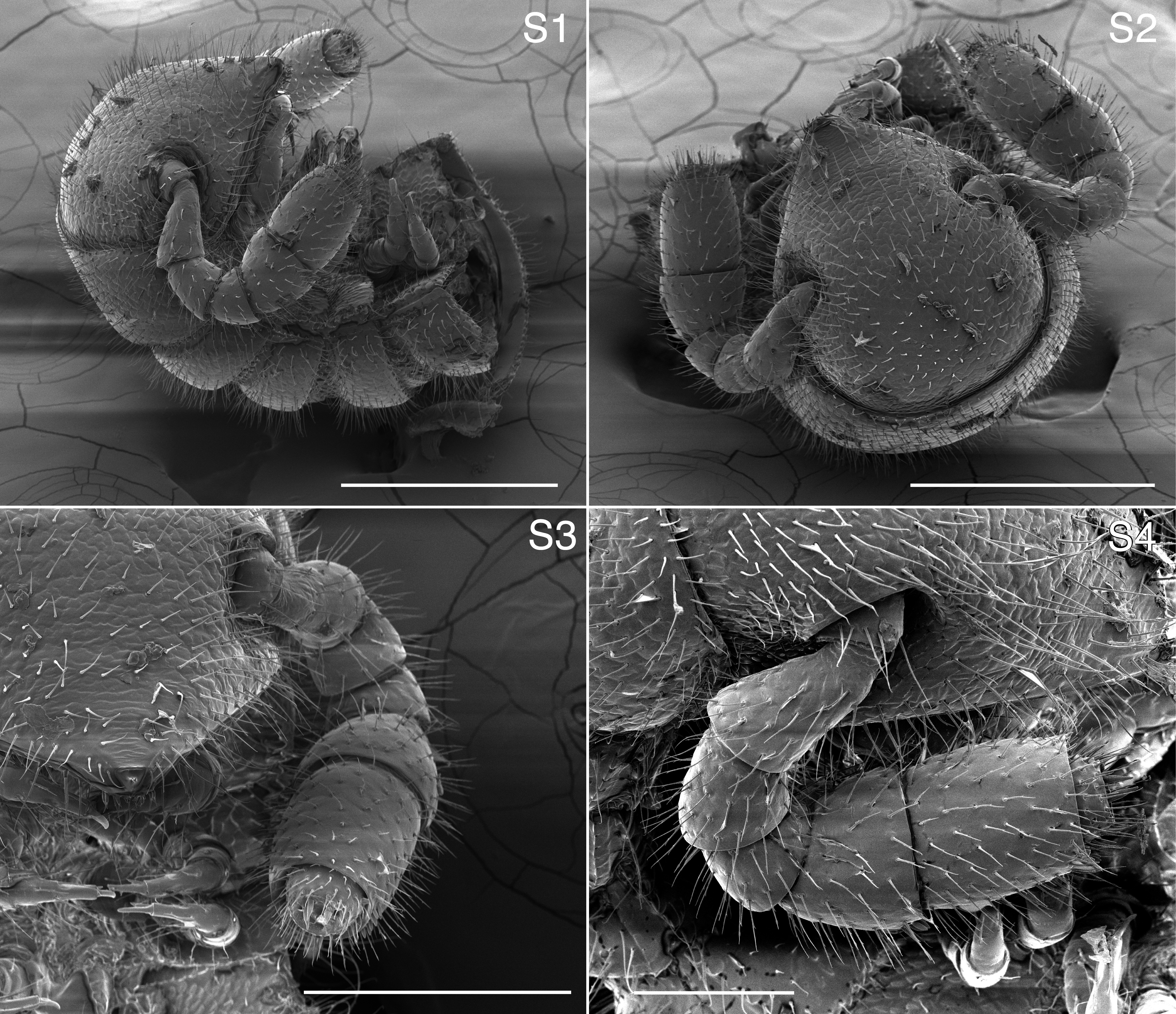

Supplement: Supplementary material 1 — Illacmesocal sp. nov. Scanning electron micrographs S1–S4 [file zookeys-1167-265_article-102537__-s001.jpg]

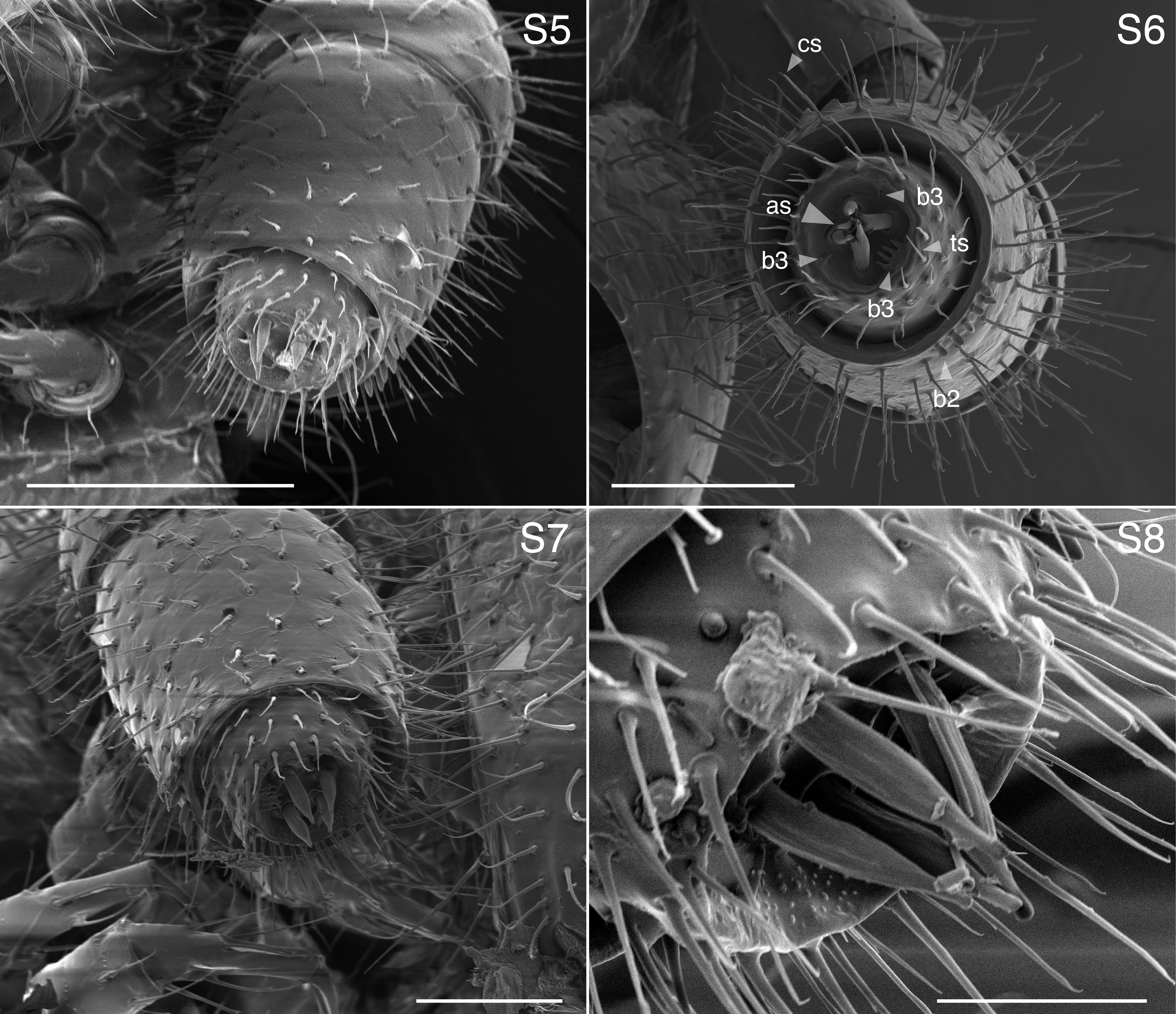

Supplement: Supplementary material 2 — Illacmesocal sp. nov. Scanning electron micrographs S5–S8 [file zookeys-1167-265_article-102537__-s002.jpg]

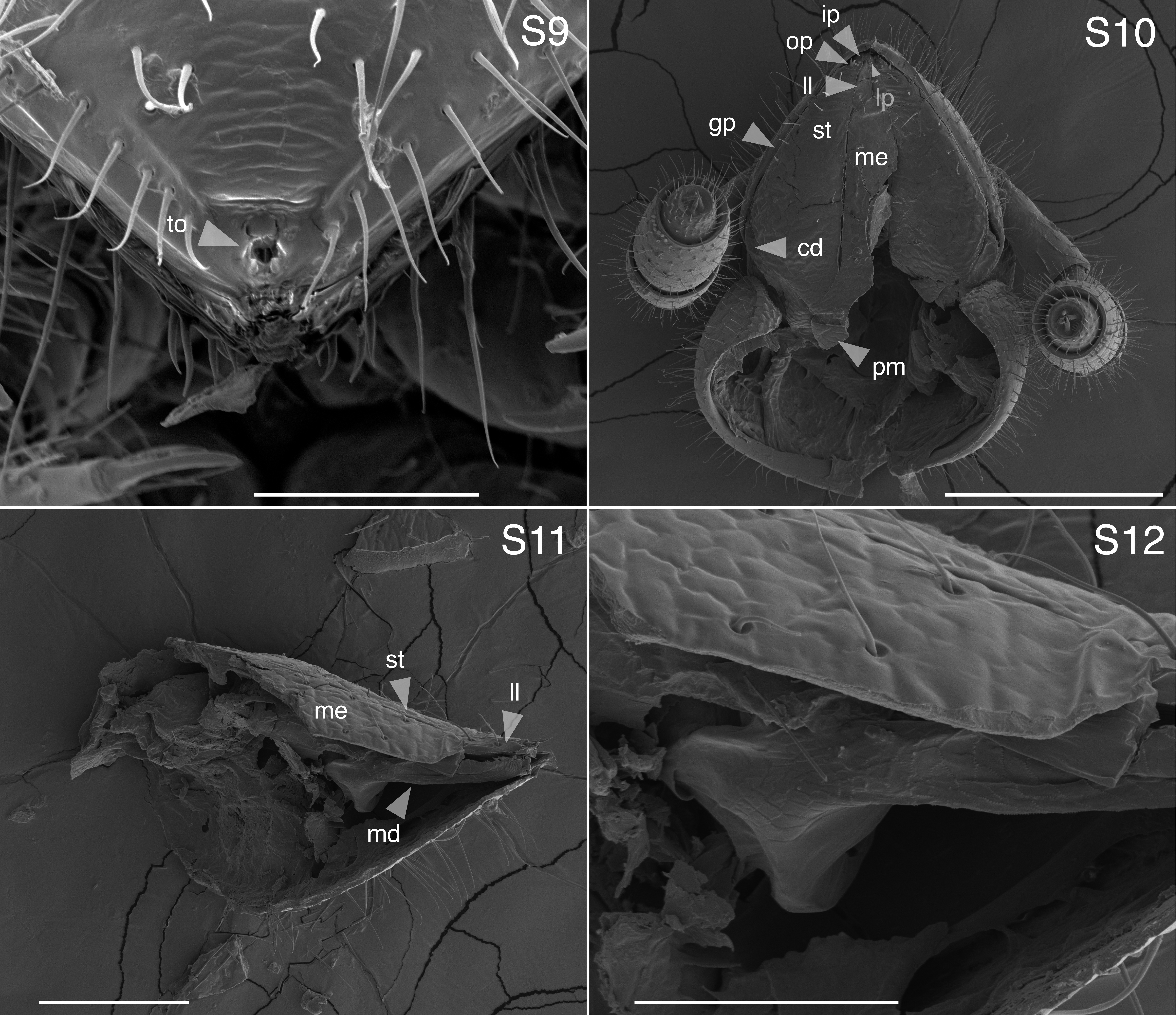

Supplement: Supplementary material 3 — Illacmesocal sp. nov. Scanning electron micrographs S9–S12 [file zookeys-1167-265_article-102537__-s003.jpg]

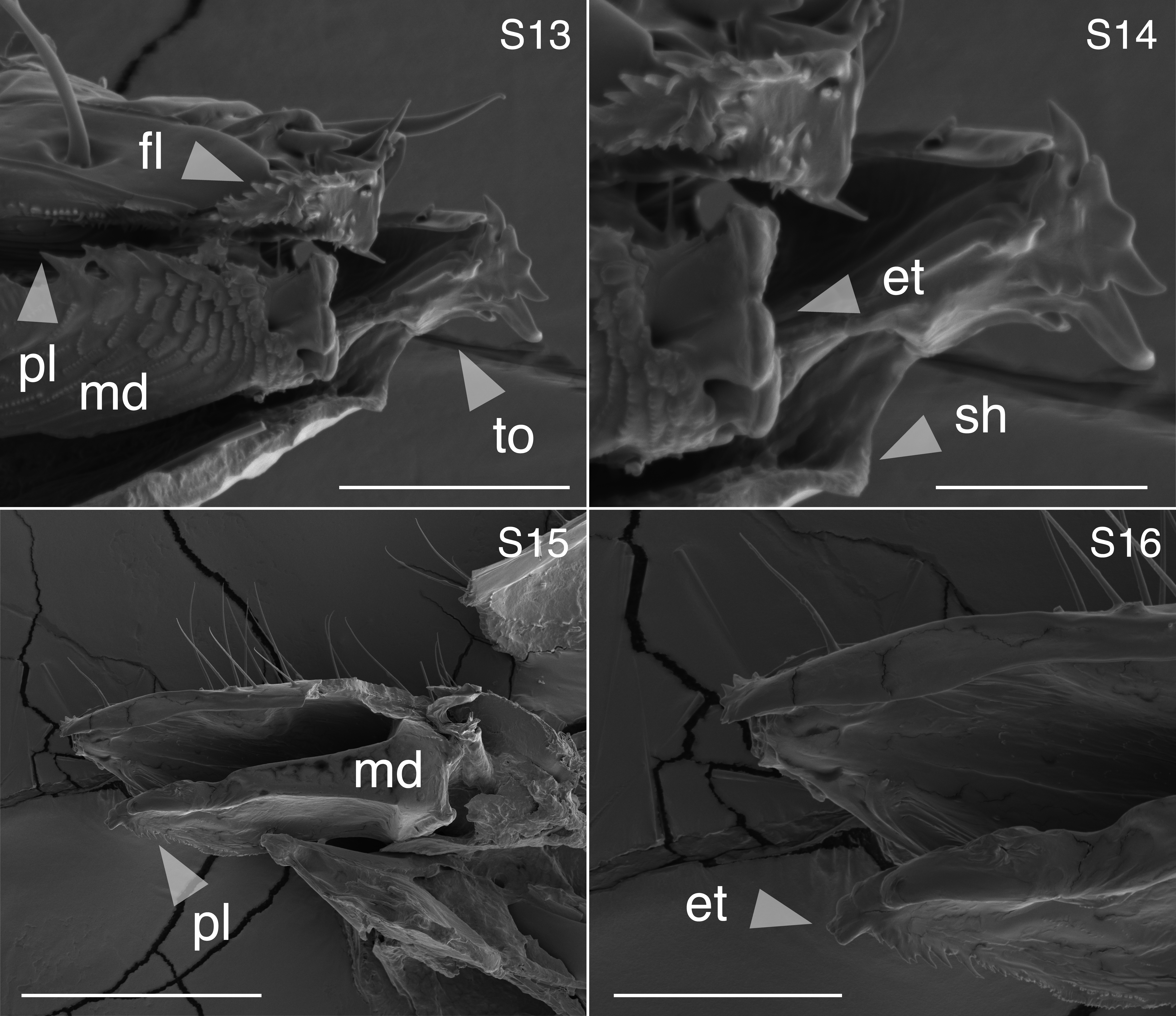

Supplement: Supplementary material 4 — Illacmesocal sp. nov. Scanning electron micrographs S13–S16 [file zookeys-1167-265_article-102537__-s004.jpg]

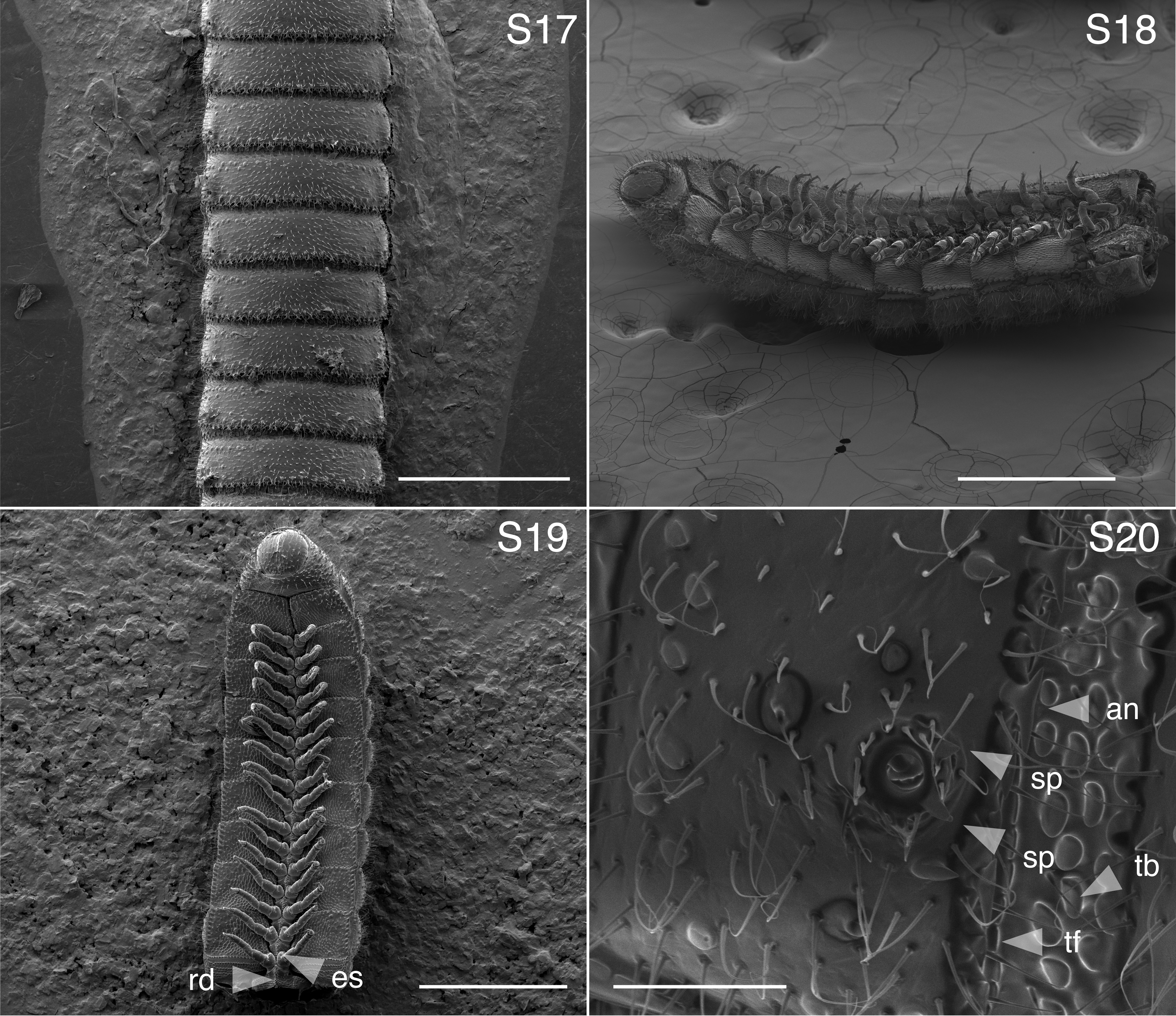

Supplement: Supplementary material 5 — Illacmesocal sp. nov. Scanning electron micrographs S17–S20 [file zookeys-1167-265_article-102537__-s005.jpg]

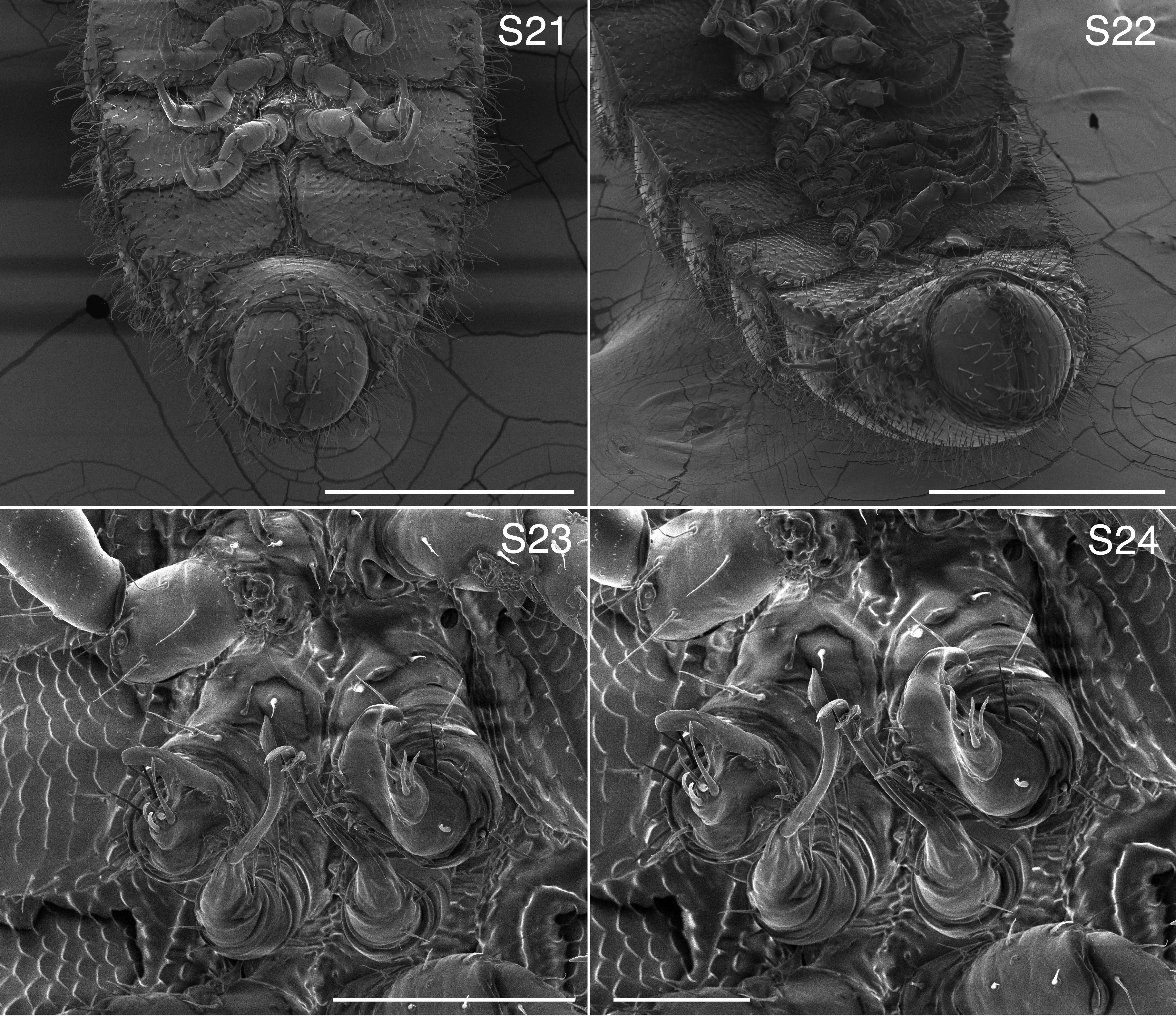

Supplement: Supplementary material 6 — Illacmesocal sp. nov. Scanning electron micrographs S21–S24 [file zookeys-1167-265_article-102537__-s006.jpg]

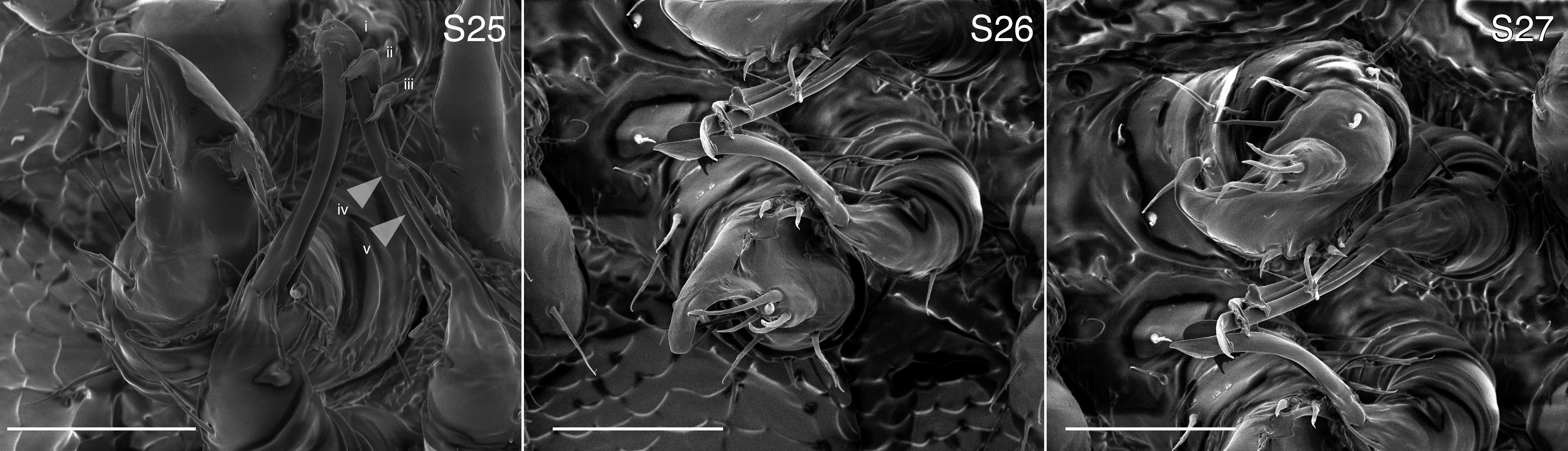

Supplement: Supplementary material 7 — Illacmesocal sp. nov. Scanning electron micrographs S25–S27 [file zookeys-1167-265_article-102537__-s007.jpg]

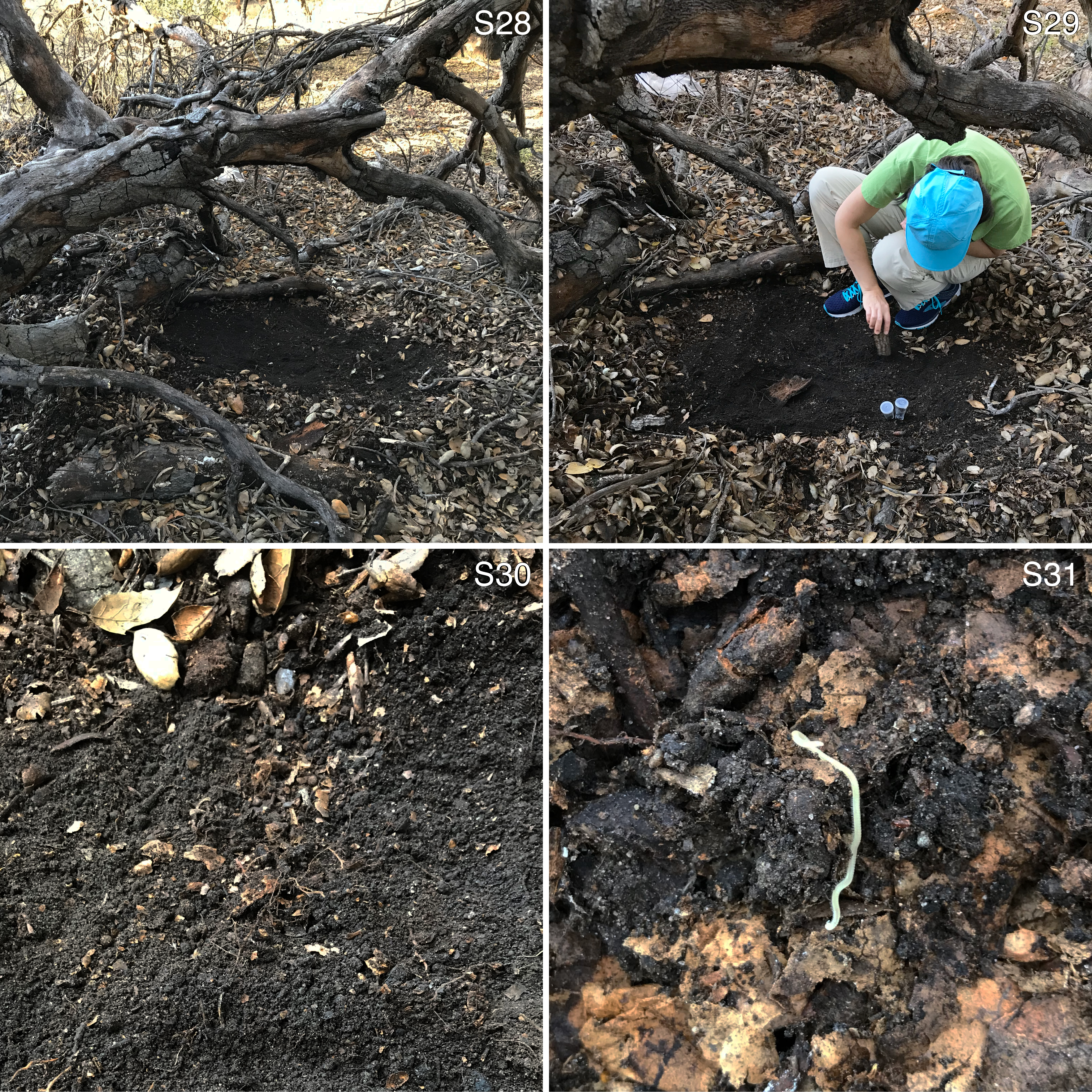

Supplement: Supplementary material 8 — Habitat of Illacmesocal sp. nov. Whiting Ranch Wilderness Park, Orange County, California [file zookeys-1167-265_article-102537__-s008.jpg]

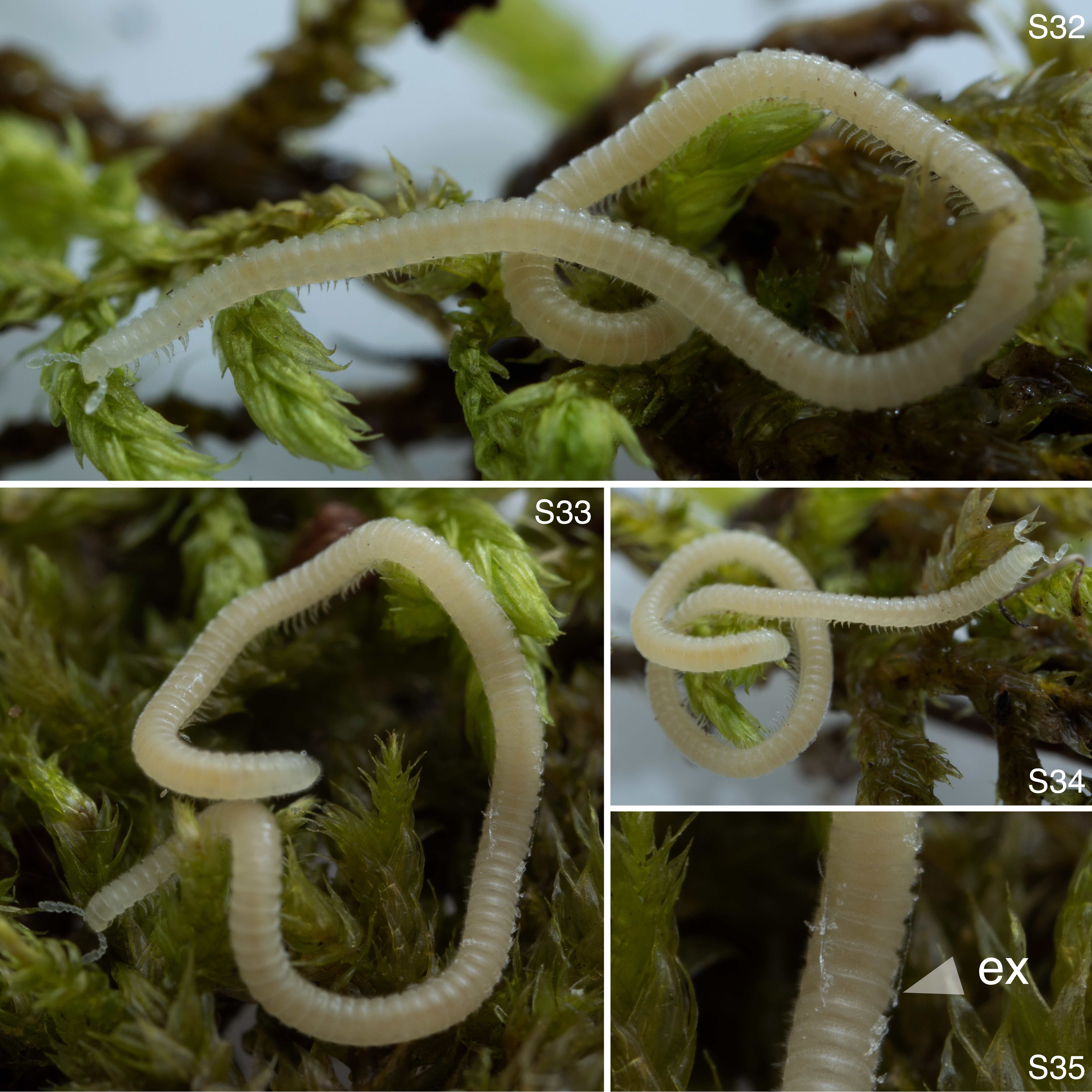

Supplement: Supplementary material 9 — Illacmesocal sp. nov. live habitus photographs S32–S35 [file zookeys-1167-265_article-102537__-s009.jpg]

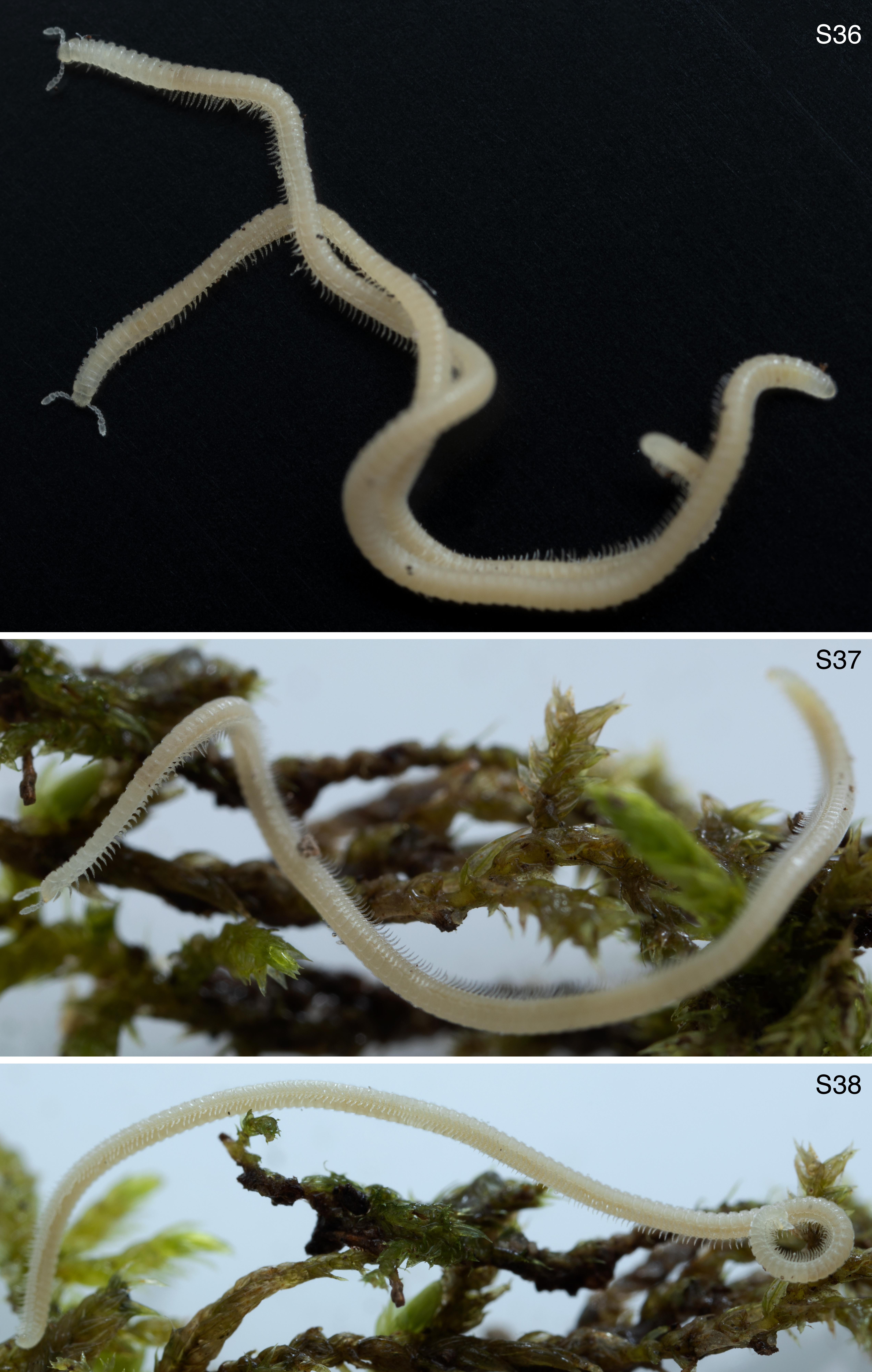

Supplement: Supplementary material 10 — Illacmesocal sp. nov. live habitus photographs S36–S38 [file zookeys-1167-265_article-102537__-s010.jpg]

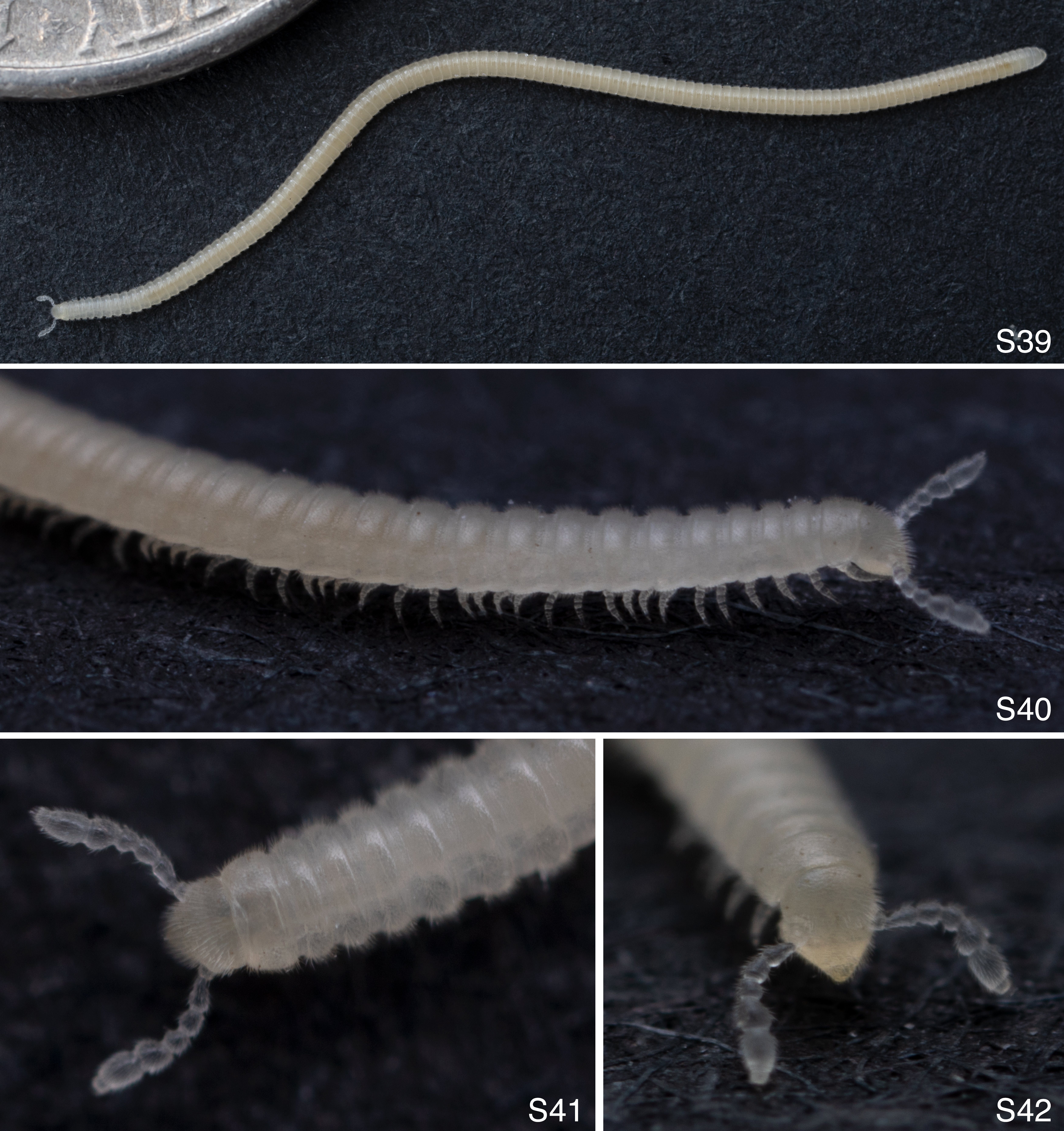

Supplement: Supplementary material 11 — Illacmesocal sp. nov. live habitus photographs S39–S42 [file zookeys-1167-265_article-102537__-s011.jpg]
